# Modeling the evolution dynamics of exon-intron structure with a general random fragmentation process

**DOI:** 10.1186/1471-2148-13-57

**Published:** 2013-02-28

**Authors:** Liya Wang, Lincoln D Stein

**Affiliations:** 1 Cold Spring Harbor Laboratory, Cold Spring Harbor, NY 11724, USA; 2 Ontario Institute for Cancer Research, 101 College St. Suite 800, Toronto, ON M5G0A3, Canada; 3 Department of Molecular Genetics, University of Toronto, 1 King's College Circle, Toronto, ON M5S 1A8, Canada

**Keywords:** Evolution of exon-intron structure, General random fragmentation process, Simulation

## Abstract

**Background:**

Most eukaryotic genes are interrupted by spliceosomal introns. The evolution of exon-intron structure remains mysterious despite rapid advance in genome sequencing technique. In this work, a novel approach is taken based on the assumptions that the evolution of exon-intron structure is a stochastic process, and that the characteristics of this process can be understood by examining its historical outcome, the present-day size distribution of internal translated exons (exon). Through the combination of simulation and modeling the size distribution of exons in different species, we propose a general random fragmentation process (GRFP) to characterize the evolution dynamics of exon-intron structure. This model accurately predicts the probability that an exon will be split by a new intron and the distribution of novel insertions along the length of the exon.

**Results:**

As the first observation from this model, we show that the chance for an exon to obtain an intron is proportional to its size to the 3rd power. We also show that such size dependence is nearly constant across gene, with the exception of the exons adjacent to the 5^′^ UTR. As the second conclusion from the model, we show that intron insertion loci follow a normal distribution with a mean of 0.5 (center of the exon) and a standard deviation of 0.11. Finally, we show that intron insertions within a gene are independent of each other for vertebrates, but are more negatively correlated for non-vertebrate. We use simulation to demonstrate that the negative correlation might result from significant intron loss during evolution, which could be explained by selection against multi-intron genes in these organisms.

**Conclusions:**

The GRFP model suggests that intron gain is dynamic with a higher chance for longer exons; introns are inserted into exons randomly with the highest probability at the center of the exon. GRFP estimates that there are 78 introns in every 10 kb coding sequences for vertebrate genomes, agreeing with empirical observations. GRFP also estimates that there are significant intron losses in the evolution of non-vertebrate genomes, with extreme cases of around 57% intron loss in *Drosophila melanogaster*, 28% in *Caenorhabditis elegans*, and 24% in *Oryza sativa*.

## Background

Most eukaryotic genes contain spliceosomal introns, which are removed from mRNA after transcription by the RNA splicing apparatus. The biological origins of introns are uncertain. Since the discovery of introns, there has been significant debate as to whether introns in modern-day organisms were inherited from a common, ancient ancestor, the intron-early hypothesis [1-3], or whether they appeared in genes more recently in the evolutionary process, the intron-late hypothesis [4,5], or indeed whether they result from a mixed model [6,7]. The mixed model suggests that most introns were gained very early in the evolution of eukaryotic genes, followed by intron loss/gain during the course of eukaryotic diversification. The details of such processes, however, remain elusive.

One way to understand the process is to examine the size distribution of internal translated exons, referring to exons that are fully translated and referred to as *itexon* in [8]. However, to avoid introducing an unfamiliar term, we will simply refer to itexons as “exon” within this communication unless specified. Although the distribution of exons is just a snapshot of the present day world, fitting it to a model based on well characterized mathematical functions may provide insights into the evolution of exon-intron structure. In a previous study, Gudlaugsdottir et al. [9] have suggested that the size distribution of exons can be approximated with a combination of Weibull [10] and exponential distributions. The Weibull distribution is a particular case of the generalized extreme value distribution. It is widely used in survival analysis, describing the size of particles generated by grinding, milling and crushing operations, etc. The exponential distribution can be used to describe the length of intervals between uniformly distributed points. Therefore, GJudlaugsdottir et al. hypothesized that the exponential distribution is the outcome of random insertion of introns (intron-late). However, they then related the Weibull distribution to the intron-early theory without providing a stochastic model that explains the observed distribution.

In later work, Ryabov and Gribskov [11] showed that a combination of two lognormal distributions gives the best fit quality to the size distribution of exons. The lognormal distribution could result from a random Kolmogoroff fractioning process [12], which assumes that the chance of fragmentation is independent of exon size. Inserting an intron into an exon is equivalent to fragmentation (splitting) of the exon. Therefore, they hypothesized that the process of intron insertion is independent of exon size.

On the other hand, Tenchov and Yanev [13] demonstrated that the Weibull distribution could result from a uniform random fragmentation process. Here, “uniform” means that the chance of fragmentation is linearly proportional to the size of the particle (or exon). Under certain conditions, the resulted Weibull distribution is indistinguishable from lognormal distribution. Hence they concluded that the model of random fragmentation could not be inferred based on the basis of fit quality. Therefore, the hypothesis that intron insertion is independent of exon size is debatable.

One assumption made in the exon size based approaches [9,11] is that introns are inserted into exon randomly. The notion of random insertion of intron has also been proposed based on the analysis of intron distribution in ancient paralogs [14]. Others have argued that there exist certain favored sites for intron insertion - the so-called proto-splice sites [4,15,16]. Unfortunately, none of the size-based approaches provide evidence to support the assumption of random intron insertion.

In this work, we aim to revisit these competing hypotheses by addressing the following open questions: Do longer exons have an increased chance of gaining a new intron? For intron gain events, will the intron be inserted into exon randomly or at some proto-splice sites? Is there an intron gain/loss bias? Are intron insertion events independent of each other? Is there a common mechanism to explain intron gain/loss in different species? In order to answer these and other related questions, we propose a General Random Fragmentation Process (GRFP) to characterize the evolution dynamics of exon-intron structures. The parameters of GRFP are determined by combining simulation and analysis of real genomic data.

## Methods

### GRFP model

The model of GRFP is motivated by generalizing both Kolmogoroff fractioning process and the uniform random fragmentation process. In GRFP, the probability for an exon to split (gaining an intron) is assumed to be exponentially proportional to the length of the *k*-th exon (*L*_*k*_) as *L*_*k*_^*a*^. Under such a generalization, the Kolmogoroff fractioning process, in which insertion events are independent of exon length, is a particular case of GRFP with *α* = 0, while the uniform random fragmentation process, in which insertions are linearly proportional to exon length, is another special case with *α* = 1. The generalization not only allows GRFP to model either Kolmogoroff or uniform fragmentation process but also allows it to model the fragmentation process of exons (intron gain) with varying α. In the results section, we will use the empirical size distribution of exons to determine the value of *α*. GRFP also assumes that introns insert into exons randomly and independently, and these assumptions are confirmed by the analysis of real genome data.

**Figure 1:**
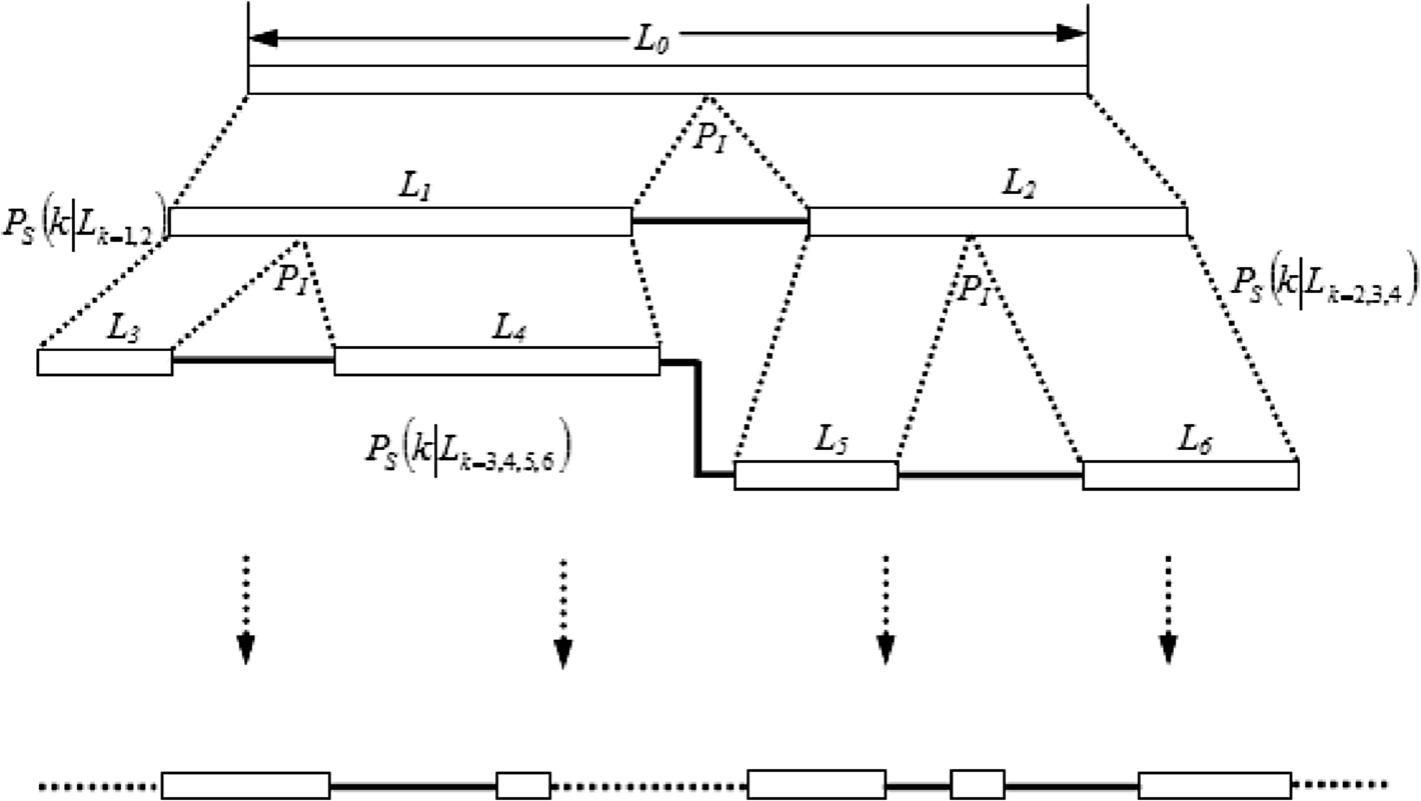
**Demonstration of GRFP on splitting a long exon with initial size *L***_**0**_**.** The probability of picking which exon to split is proportional to the length of the exon, *P*_S_(*k*) ~ *L*_*k*_^*α*^. The probability of picking an inserting point (*xє*(*O, L*_*k*_)) for exon *k* follows a normal distribution, *P*_I_(*x*) ~ *L*_k_*N(^*μ*^ _*I*_, *σ*_*I*_).

The model of GRFP, illustrated in Figure 1, is summarized below:

1. Given a set of *n* exons, the opportunity for *k*-th exon to acquire a new intron is proportional to its size to the *α*-th power:

(1) $$\;P_{s}\left(K\right)=L_{k}^{a}/\sum_{k=1}^{n}L_{k}^{a}$$

2. Within *k*-th exon, the new intron insertion loci follow a normal distribution:

(2) $$\;P_{I}\left(x\right)\sim L_{k}*N\left(\mu_{I},\sigma_{I}\right),x\in \left(\begin{array}{ll} 0, & L_{k} \end{array}\right)$$

3. Intron gains are independent of each other.

Where *Р*_*Ѕ*_*(K)* denotes the probability for *k*-th exon to obtain an intron; *Р*_*I*_*(x)*, probability of inserting an intron after *x*-th position within *k*-th exon; *L*_*k*_, length of the exon *k*; *α*, dependency value; *μ*_*I*_ and *σ*_*I*_, mean and standard deviation of the distribution of insertion loci. The model of GRFP has three unknown parameters to be determined, *α*, *μ*_*I*_ and *σ*_*I*_.

### Simulation testing

We start each simulation with a long exon. The diagram in Figure 1 shows how the splitting of a long exon during evolution is simulated. The diagram shows intron gains in the following order. After inserting the first intron, *Ρ*_*Ѕ*_*(K|L*_*k*=1,2_) denotes the picking probability between *L*_1_ and *L*_2_; Assuming *L*_1_ is selected and split by a new intron; the next exon to be split will be chosen from *L*_3_, *L*_4_ and *L*_2_ with probability *Ρ*_*Ѕ*_*(K|L*_*k*=2,3,4_); Assuming *L*_2_ is selected, and so on.

For simulations, sequence of pseudorandom number is obtained using the *Mersenne Twister* algorithm [17] implemented in standard MATLAB 7.12. In this study, all of the simulations start with one single exon. This simplifies the simulation, but it does not imply that the evolution of eukaryotic genes always starts with one long exon. Under such simplification, two more parameters are the initial size of starting exon (*L*_0_) and the number of splitting (*m*). For a given species, we can estimate them from the annotated gene sets.

We evaluate the properties of GRFP using three simulation experiments. In each, we simulate a set of ordered fragments and quantify their statistical characters given different parameters. The three sets of quantifications listed below are used to justify the three assumptions of GRFP respectively for both simulated fragments and real exons.

1. Mean and standard deviation of the size distribution by fitting it with lognormal distribution (equation (3)) or Weibull distribution (equation (4)):

(3) $$\;\;\mathrm{dN}=\left(N/\left(\sigma_{E}\sqrt{2\pi }\right)e^{{-\left(\left(1\mathrm{nE}-\mu_{E}\right)/\sqrt{2\sigma_{E}}\right)}^{2}}\right)d\;\text{ln}E$$

(4) $$\;\;\mathrm{dN}=\left({\mathrm{Nkz}}^{k-I}e^{-zk}/\lambda \right)d\;\text{ln}E$$

Where z=E/λ, *E* is exon length, *dN* the number of exons in a bin (bin size is 0.1 unless specified), *N* the amplitudes of the peak; *k* shape parameter, and *λ* scale parameter of the Weibull distribution; *μ*_*E*_ the mean position, and σ_*E*_ the standard deviation of the lognormal distribution. These and subsequent fittings in this study are performed using the nonlinear Trust-Region-Reflective curve-fitting algorithm [18,19] implemented in MATLAB 7.12. Simulation demonstrates that σ_*E*_ is primarily determined by the choice of *α* (in equation (1)).

2. Mean and standard deviation of the insertion ratio defined below:

(5) $$\;x_{i}=L_{i}/\left(L_{i}+L_{i+1}\right)$$

Where *L*_*i*_ and *L*_*i+1*_ are the length of two adjacent fragments (exons). This is an indirect estimation of insertion loci (*x* in equation (2)). Both simulation and real genome data indicate that *x* follows a normal distribution (with a standard deviation *σ*_*x*_).

3. Correlation between *x*_*i*_ and *x*_*j*_ defined by equation (6):

(6) $$\;\rho \left(i,j\right)=\frac{\sigma_{x_{i}+x_{j}}^{2}-\sigma_{x_{i}}^{2}-\sigma_{x_{j}}^{2}}{2\sigma_{x_{i}}\sigma_{x_{j}}}$$

Where *σ*_*x*_ is estimated from fitting the histograms of ratio *x* with a normal distribution. In theory, *x*_*i*_ + *x*_*j*_ is still normally distributed, and the mean value is the sum of the means. However, the variances are not additive if *x*_*i*_ and *x*_*j*_ are correlated. We can estimate the relationship between *x*_*i*_ and *x*_*j*_ with equation (6).

In the first experiment, we examine the relationship between GRFP parameters and the size distribution of the simulated fragments. With fixed *μ*_*I*_, *σ*_*I*_, initial size of starting exon (*L*_0_), and the number of splitting (*m*), one long exon is fragmented with different choices of *α*. *μ*_*E*_ and *σ*_*E*_ are estimated through fitting a lognormal distribution to the size distribution of the resulted fragments. The correlation between *μ*_*E*_, *σ*_*E*_ and *α* is examined. Then, with fixed *μ*_*I*_, *σ*_*I*_, and *α*, the relationship between *μ*_*E*_, *σ*_*E*_ and initial size of starting exon (*L*_0_), the number of splitting (*m*) is examined through similar simulations.

In the second experiment, we examine the relationship between real *σ*_*I*_ (in equation (2)) and estimated *σ*_*x*_ (from equation (5)). By fragmenting a long exon, we construct a binary tree to track the splitting process. We classify the adjacent fragments pair (the order is maintained during fragmentation) into four groups based on whether they have the same parent nodes, or if not same parents, comparing their depths. The size distribution of each group and the mixture (equation (5)) is examined. With fixed *μ*_*I*_, *L*_0_, *m*, and *α*, the correlation between *σ*_*I*_ and *σ*_*x*_ is examined by simulations with different choices of *σ*_*I*_. Then, by coupling with empirical observations, we use Expectation-Maximization (EM) iteration to determine the value of *α* and *σ*_*I*_.

In the third experiment, we examined the effects of intron loss on the statistical characters of resulted fragments. By introducing various percentages of intron loss after intron gain, we evaluate how *σ*_*E*_, *σ*_*x*_, and *ρ(i,j)* of the resulted fragments are changed.

### Empirical data analysis

In this study, we obtained the cDNA sequences of 14 species (*Homo sapiens* (GRCh37.p8), *Mus musculus* (GRCm38), *Rattus norvegicus* (RGSC3.4), *Danio rerio* (Zv9), *Caenorhabditis elegans* (WBcel215), *Drosophila melanogaster* (BDGP5), *Bos taurus* (UMD3.1), *Pan troglodytes* (CHIMP2.1.4), *Gallus gallus* (WASHUC2), *Sus scrofa* (Sscrofa10.2), *Arabidopsis thaliana* (TAIR10), *Oryza sativa* (MSU6), *Sorghum bicolor* (Sorbi1), *Zea mays* (AGPv2)) from Ensembl and plant Ensembl database [20]. To ensure the quality of the data, we only use the cDNA sequences of protein coding genes with both RefSeq mRNA ID and the known status of both gene and transcript. To examine the size distribution of exons, we extracted the genomic positions of the exons from cDNA sequences to compute exon sizes. We also extracted the genomic positions of the 5′ and 3′ UTRs and used them to identify internal translated exons.

For testing the first assumption of GRFP, we fitted both Weibull and normal distribution to the size distribution of vertebrate exons (logarithm scale). We also grouped exons by positions for testing position bias of intron gain/loss. For the second assumption, we fitted a normal distribution to *x* (equation (5)). For the third assumption, we computed *ρ(i,j)* for exon pairs at *i*-th and *j*-th position in all protein coding genes. Finally, we examined the differences in the parameters fitted to vertebrate and non-vertebrate species.

## Results

### Empirical data analysis

#### Statistical counts of empirical data

Statistical counts of the extracted data are shown in Table 1. The transcript with the longest CDS (Coding DNA Sequence) for each gene is used for counting the number of protein coding genes, number of splitting, and total CDS length. In these counts, a coding gene is excluded if it does not contain any internal translated exons. The total CDS length is the summation of the length of all exons. The number of splitting events is the total number of exons minus one (for reversion of splitting, a long exon can be reconstructed by connecting all exons together). The last two columns of Table 1 are estimated through GRFP simulations that will be discussed later.

**Table 1:** Statistical counts of coding genes, splitting (number of exons minus one) and total CDS length (b.p.)

	**Number of coding genes**	**Total CDS length (10**^**7**^**)**	**Number of splitting (*m, *** **10**^**5**^**)**	**Estimated splitting (*m***_**e**_**, 10**^**5**^**)**	$\frac{m-m_{e}}{m_{e}}$
*H*. *sapiens*	17275	2.443	1.827	1.901	- 3.9%
*M*. *musculus*	16319	2.276	1.705	1.768	- 2.7%
*R*. *norvegicus*	17354	2.193	1.722	1.703	1.0%
*D*. *rerio*	15068	1.932	1.462	1.501	- 2.7%
*G. gallus*	5416	0.655	0.537	0.509	5.4%
*P. troglodytes*	12508	1.694	1.295	1.316	- 1.6%
*B. taurus*	11948	1.413	1.142	1.099	4.0%
*S. scrofa*	5498	0.524	0.433	0.408	6.1%
*C. elegans*	17684	1.833	1.024	1.425	- 28.4%
*D. melangaster*	8063	1.141	0.383	0.886	- 57.1%
*A. thaliana*	16547	1.501	1.083	1.167	- 7.2%
*O. sativa*	23566	2.255	1.329	1.749	- 24.0%
*S. bicolor*	17769	1.445	1.041	1.123	- 7.3%
*Z. mays*	15887	1.320	0.987	1.025	- 3.7%

Annotation data for each species is extracted from Ensembl database. Protein coding genes are counted only if they contain at least one internal translated exon. Total CDS length is the summation of all internal translated exon length in these genes. Number of splitting is estimated by the number of internal translated exons minus one. Estimated splitting is determined from GRFP simulation.

In this study, we ignored non-internal translated exons considering the rate of indels (a type of mutations affecting exon size distribution) is significantly lower in the coding region than the non-coding region [21]. It is true that introns can be inserted anywhere, including non-coding exons or even another intron, but their size distribution is confounded by the appearance of more frequent indels.

### Size distribution of exons

**Figure 2:**
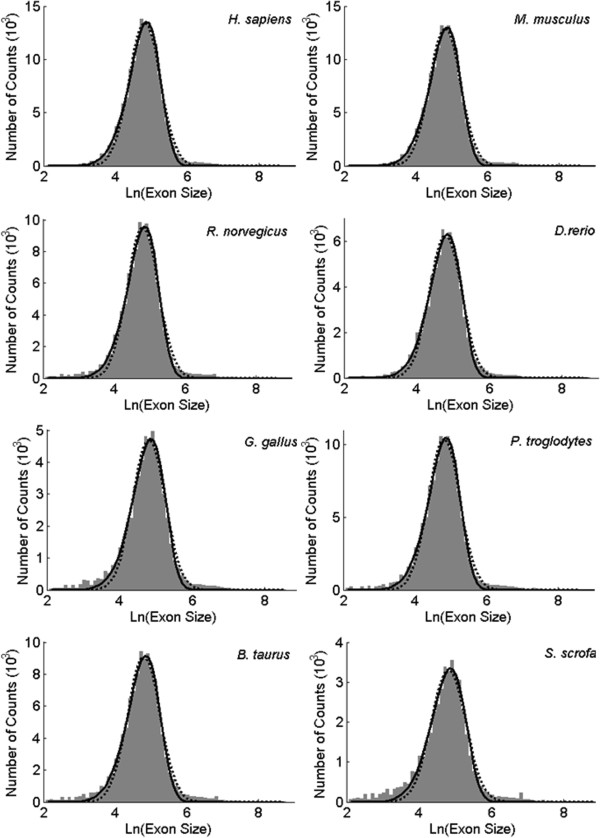
**Size distributions of vertebrate exons fitting with normal distribution.** The histograms of exons are fitted with a Weibull function (solid line) and normal function (dashed line).

Figure 2 shows the histograms of exons for eight vertebrate species. Both Weibull (solid line) and normal (dashed line) functions provide a reasonable fit to the histograms of exons, with the fitted parameters shown in Table 2. Notably, in Table 2, the fitted parameters are almost identical across species (e. g., *μ*_*E*_ and *σ*_*E*_), which might indicate that these vertebrate genomes have undergone a similar stochastic process on the exon-intron structure during evolution. For the six non-vertebrate species, a mixture of two normal functions (dashed line) fits the histograms well (Additional file 1: Figure S1). This might suggest that the evolution of non-vertebrate exon-intron structure has undergone other processes.

**Table 2:** Fitted parameters for size distributions of observed vertebrate exons

	**Weibull**		**Normal**	
	***λ***	***κ***	***μ***_***E***_	***σ***_***E***_
*H*. *sapiens*	2.81	6.98	4.81	0.432
*M*. *musculus*	2.81	7.01	4.82	0.431
*R*. *norvegicus*	2.80	6.89	4.81	0.437
*D*. *rerio*	2.79	6.87	4.79	0.437
*G. gallus*	2.80	6.80	4.81	0.442
*P. troglodytes*	2.81	6.87	4.81	0.440
*B. taurus*	2.80	6.69	4.80	0.449
*S. scrofa*	2.82	6.44	4.81	0.472

The size distribution of exon (logarithmic scale) for each vertebrate species is shown in Figure 2. Each distribution is fitted with equation (3) and (4) separately. The normal function fitting is shown as dashed line. The Weibull function fitting is shown as a solid line.

**Figure 3:**
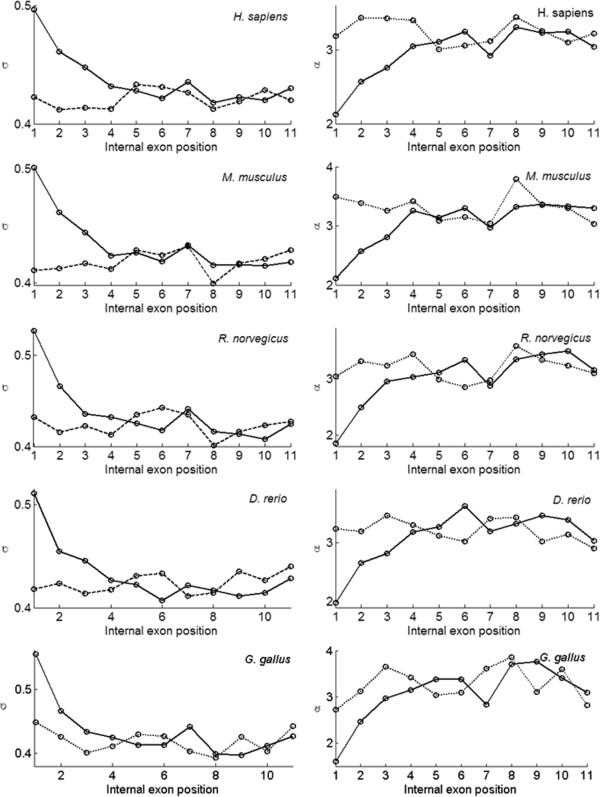
**Fitted standard deviation (*σ***_***E***_**) and dependency (α) for internal exons with positions relative to 5′ UTRs (solid line) or 3′ UTRs (dashed line).** The dependency value α is calculated using equation (9).

In order to assess whether the size distribution of vertebrate exons is position-dependent, we grouped their exons from all protein coding genes according to their positions relative to 5′ UTRs/3′ UTRs. For the five well annotated vertebrates, the standard deviations (*σ*_*E*_) of the fitted normal functions at each position (e.g. Additional file 1: Figure S2 for *H. sapiens*) are shown in the left panel of Figure 3. The right panel shows corresponding *α* values calculated using equation (9). The mean values of these distributions are almost constant at all positions (results not shown). Figure 3 shows that *σ*_*E*_ is almost constant for exons across gene body, with exceptions of the first three exons right after 5′ UTR (see solid line), where it increases markedly. For exons next to the 3′ UTR (in dashed line), no similar trend is observed.

These observations suggest that the size distribution of vertebrate exons could be properly fit with either Weibull or normal distribution. The Weibull distribution gives a better fit to both left and right tails (e.g., Additional file 1: Figure S2) because the distribution is skewed to the left. For numerical simulations, we will show that similar size distribution of fragments will be generated based on GRFP model.

### Distribution of insertion ratio

**Figure 4:**
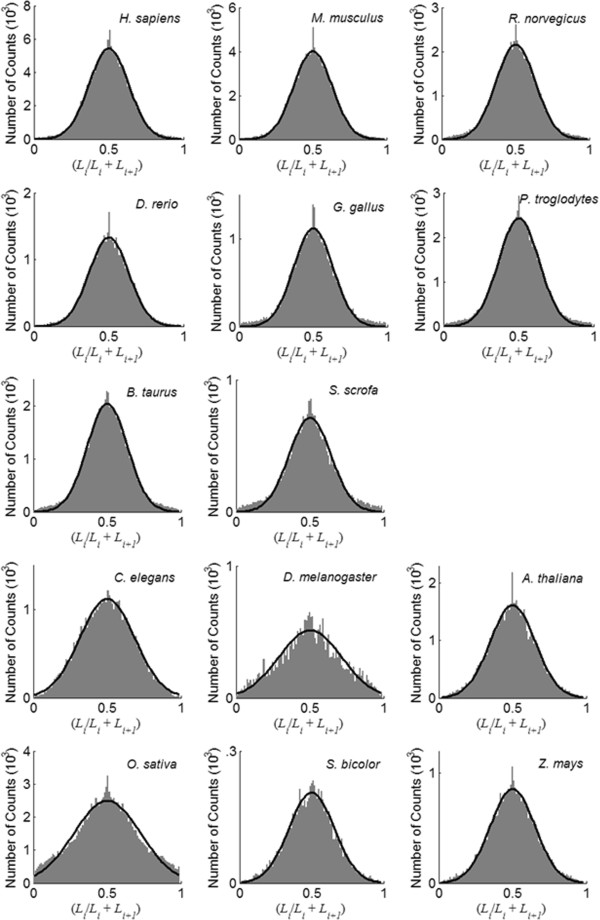
**Genome wide distribution of *L***_***i***_**/(*L***_***i***_**+ *L***_***i*+1**_**).** The histograms are drawn with bin size of 0.01, and fitted with a Normal function.

For every gene of the selected species, we calculated the insertion ratio *x* (equation (5)) for each adjacent exon pairs *L*_i_ and *L*_i+1_. Figure 4 shows the histograms of the ratios for the 14 species. The histograms are fitted well with a normal distribution, and the fitted parameters are shown in Table 3. The fitted parameters are almost identical across vertebrate species with *μ*_*x*_= 0.5 and *σ*_*x*_= 0.13. The insertion ratio for non-vertebrates fits a normal distribution reasonably well but with much larger *σ*_*x*_.

**Table 3:** Fitted parameters for distribution of insertion ratios from empirical data

	***μ***_***x***_	***σ***_***x***_
*H*. *sapiens*	0.501	0.132
*M*. *musculus*	0.501	0.132
*R*. *norvegicus*	0.501	0.135
*D*. *rerio*	0.501	0.132
*G. gallus*	0.501	0.132
*P. troglodytes*	0.502	0.134
*B. taurus*	0.502	0.136
*S. scrofa*	0.502	0.142
*C. elegans*	0.499	0.185
*D. melangaster*	0.502	0.215
*A. thaliana*	0.501	0.152
*O. sativa*	0.502	0.226
*S. bicolor*	0.501	0.157
*Z. mays*	0.501	0.152

The distribution of insertion ratios (equation (5)) for each species is shown in Figure 4. Each distribution is fitted with a normal distribution (equation (3)).

Another interesting observation in Figure 4 is the sharp spike at 0.5, which suggests that there are excessive adjacent exons pairs with the same length. This is consistent with the observation of tandem exon duplication [22]. Because the spikes are located right on the center, mathematically such deviation has little effect on the fitting of the histogram.

The normal function fitted in Figure 4 describes where introns get inserted into an exon. To assess whether it is consistent or against the hypothesis of proto-spliced sites, we calculate the position distribution of four possible proto-splice sites (tested in [23,24]) within human coding sequences, and the results are shown in Additional file 1: Figure S3. The position for each of the four sites (G|G, AG|G, AG|GT and (C/A)AG(A/G)) is calculated by dividing the distance between the intron starting site and start codon by the length of the coding sequence. All coordinates are extracted from Ensembl annotation of *H. sapiens*. The symbol “|” stands for the intron position and “/” indicates wo alternative states of one nucleotide site. Additional file 1: Figure S3 shows that these proto-spliced sites are distributed nearly uniformly within CDS. If introns strongly prefer to be inserted into these sites, the insertion ratio should follow a uniform distribution instead of normal distribution as we observed. Therefore, the analysis here does not favor the proto-splice site hypothesis.

### Correlation between insertion ratios

**Figure 5:**
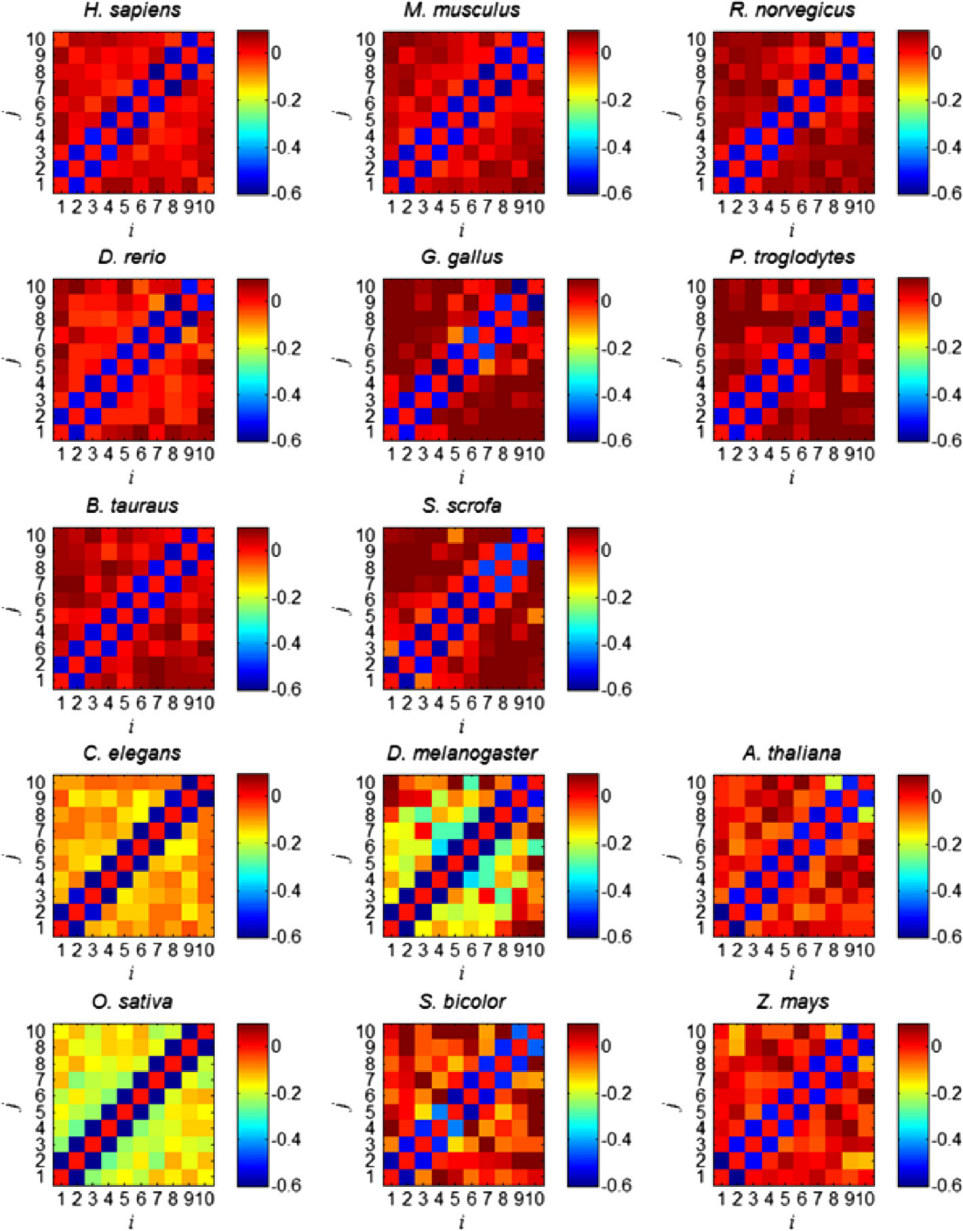
**Correlation of insertion ratios for different species.** The correlation between *x* and itself is set to zero (the diagonal blocks). The color bar on the right indicates the correlation value between insertion ratio and ratios with *i* or *j* distance to it.

The correlations calculated using equation (6) are shown in Figure 5. For better visualization purpose, we set the correlation between *x* and itself to zero (the diagonal blocks). The color bar on the right indicates the correlation value between insertion ratio and ratios with *i* or *j* distance to it. Figure 5 shows that *ρ*(*i*, *j*) is significantly negative if |*i* - *j*| = 1. While *ρ*(*i*, *j*) is close to zero for vertebrates if |*i* - *j*| > 1. The negative correlation between adjacent insertion ratio is not surprising since the calculation uses the same exon length as dividend (equation (5)) but with opposite signs. For example, considering three exons in order with length *p*, *L*-*p*, and *q*, the insertion ratios are *x*_1_*= p/*(*p+L-p*) *= p*/*L* and *x*_2_ = (*L*-*p*)/(*L*-*p*+*q*) ≈ 1-*p*/*L* if *p* ≈ *q*; Both *x*_1_ and *x*_2_ are proportional to *p* but with different signs.

The key observation in Figure 5 is that nonadjacent insertion ratios are nearly uncorrelated for vertebrate genomes. However, the correlation between intron insertion ratios has quite different patterns for non-vertebrates. Their insertion ratios are more negatively correlated with each other than those for vertebrates, especially for *C. elegans*, *D. melanogaster*, and *O. sativa*.

In summary, analysis of empirical data reveals three significant differences between vertebrate and non-vertebrate genomes. First, a mixture of two normal functions gives a better fit to the size distribution of non-vertebrate exons, instead of one normal function for that of vertebrate exons; Second, the insertion ratio of non-vertebrates also follows a normal distribution but with larger standard deviation than that of vertebrates; Third, the insertion ratios of non-vertebrates are more negatively correlated than that of vertebrates.

### Simulation testing

#### Default values for *L*_0_, *m*, *σ*_*I*_, and *μ*_*I*_

As mentioned before, we start each simulation with a long exon. Using the counts for *H. sapiens* (Table 1), we set the initial exon size and number of splitting to following values for all simulations unless specified:

(7) $$\;L_{0}=2.4\times 10^{7},m=1.8\times 10^{5}$$

For the remaining unknown parameters of GRFP, *α*, *σ*_*I*_, and *μ*_*I*_, we chose to examine *α* first with following values for *σ*_*I*_ and *μ*_*I*_:

(8) $$\;\sigma_{I}=0.11,\mu_{I}=0.5$$

These values are determined through an EM iteration process that will be discussed in the simulation testing section. The EM iteration uses observed values of *σ*_*x*_ and *μ*_*x*_ for vertebrates (Table 3). The simulation described below shows that *σ*_*x*_ overestimates but is linearly proportional to *σ*_*I*_, while *μ*_*x*_ approximates *μ*_*I*_ extremely well.

#### Relationship between α, *L*_0_, *m* and *σ*_*E*_, *μ*_*E*_

Using the values of *L*_0_, *m*, *σ*_*I*_ and *μ*_*I*_ in equations (7) and (8), we performed three GRFP simulations with *α* values of 0.3, 1 and 3. The size distributions of the GRFP fragments are shown in Additional file 1: Figure S4. Both the Weibull and normal functions were used for fitting to the logarithm size distribution. Fitted parameters are shown in Table 4. Numerically the Weibull function is unstable for fitting the histogram when *α* is close to zero. Therefore, we picked *α* = 0.3 to mimic a random Kolmogoroff fractioning process. *α* = 1 would correspond to a uniform random fragmentation process, and *α* = 3 will generate size distribution similar to that of real exons for the vertebrate genomes (in both shape and standard deviation). We found that although the Weibull function fits the tails of the distributions better, the fitted parameters for Weibull are extremely sensitive to the minimum size of the GRFP fragments. Therefore, in this study we use *σ*_*E*_ of the fitted normal function to characterize the peak width of the size distribution. It is worthwhile reemphasizing that both empirical and simulated distributions are skewed to the left; thus both tails of the peak are better fitted by the Weibull distribution.

**Table 4:** Fitted parameters for size distribution of simulated exons

	**Weibull**		**Normal**	
	***λ***	***κ***	***μ***_***E***_	***σ***_***E***_
*α* = 0.3	4.58	3.95	4.24	1.216
*α* = 1	2.88	4.42	4.72	0.688
*α* = 3	2.93	7.25	4.85	0.430

The size distribution of simulated exon (logarithmic scale) for each choice of dependency value (*α*) is shown in Additional file 1: Figure S4. Each distribution is fitted with equation (3) and (4) separately. The normal function fitting is shown as dashed line. The Weibull function fitting is shown as a solid line.

**Figure 6:**
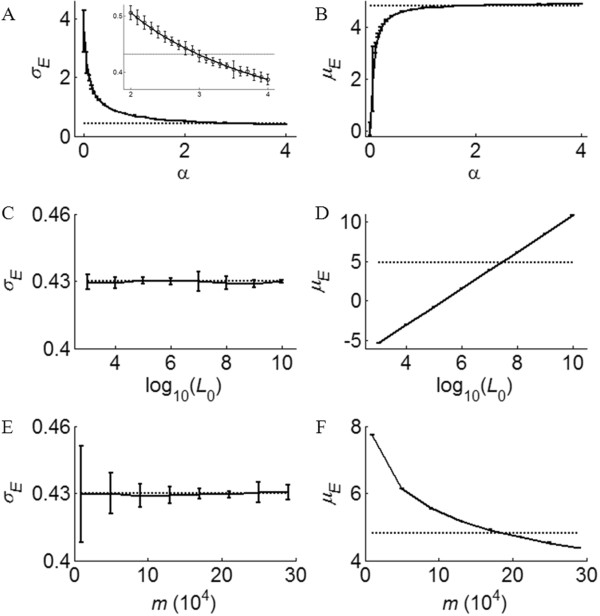
**Relationship between GRFP parameters and *σ***_***E***_**, *μ***_***E***_**.** (**A**) Plot of *σ*_*E*_ and (**B**) *μ*_*E*_ as a function of *α*; (**C**) Plot of *σ*_*E*_ and (**D**) *μ*_*E*_ as a function of *L*_*0*_); (**E**) Plot of *σ*_*E*_ and (**F**) *μ*_*E*_ as a function of *m*. 3 simulations are performed for each test and plus/minus three standard deviations are shown in vertical bars. The dashed lines show where *σ*_*E*_ and *μ*_*E*_ of *H. sapiens* are (Table 2).

These simulations show that *σ*_*E*_ (or width of the peak) decreases as *α* increases. To quantify how *σ*_*E*_ is dependent on *α*, we performed three GRFP simulations for each *α* value range from 0 to 4. The size distribution of GRFP was fitted to a normal function, and the fitted *σ*_*E*_ and *μ*_*E*_ values (mean ± 3 standard deviations) were plotted against *α* in Figure 6A. For *α* values ranging between 2 and 4, we found that the relationship between *σ*_*E*_ and *α* can be fitted with the following equation:

(9) $$\;\;\sigma_{E}=0.54/a^{0.56}+0.14$$

From equation (9), we estimate that *α* ≈ 3 gives the observed *σ*_*E*_ ≈ 0.43 (Table 2). This suggests the chance of intron gain is proportional to the exon length to the 3^rd^ power, which disagrees with the independency hypothesis of earlier work [11].

Similarly, we performed a series of GRFP simulations with different choices of *L*_0_ and *m*, and the results are shown in Figure 6C-F. Figure 6C and 6E show that the estimated *σ*_*E*_ is independent of both *L*_0_ and *m*, while Figure 6D and 6F demonstrates that the mean value (*μ*_*E*_) of the resulting size distribution is dependent on both *L*_0_ and *m*. From Figure 6F, we can estimate *m* via the GRFP simulation given *σ*_*E*_ and *L*_0_, using the intersection between the dashed line (*μ*_*E*_ of *H. sapiens*) and the solid curve. Given that *μ*_*E*_ is approximately 4.81 across vertebrate genomes, we used GRFP simulation to estimate the number of splitting (*m*_e_) for each species (Table 1). The percentage of intron loss is estimated by comparing *m*_e_ with *m*. For other species, the corresponding *L*_0_ in Table 1 is used to estimate *m*_e_. *m*_e_ and the percentages of intron loss are calculated in the same way. Note that here we use the same *μ*_*E*_ value of 4.8 for invertebrates although the size distributions of their exons (Additional file 1: Figure S1) are quite different (we hypothesize that they are resulting from intron loss). These estimations show that there are approximately 24% intron loss in *O. sativa*, 28% intron loss in *C. elegans*, and 57% intron loss in *D. melanogaster*, relative to what is predicted by GRFP model based on CDS length.

(10) $$\;m={0.0078L}_{0}+84$$

**Figure 7:**
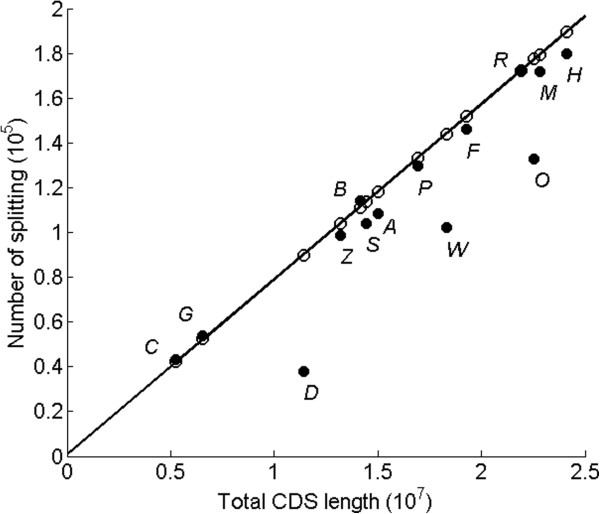
**Plot of the number of splittings as a function of total CDS length.** Estimated splitting (open circle) is from GRFP simulation with different CDS lengths (Table 1) and fits with a linear function (solid line) shown in equation (10). The observed splitting (closed circle) is also plot. Species are *Arabidopsis thaliana* (***A***), *Bos taurus* (***B***), *Sus scrofa* (***C***), *Drosophila melanogaster* (***D***), *Danio rerio* (***F***), *Gallus gallus* (***G***) *Homo sapiens* (***H***), *Mus musculus* (***M***), *Oryza sativa* (***O***), *Pan troglodytes* (***P***), *Rattus norvegicus* (***R***), *Sorghum bicolor* (***S***), *Caenorhabditis elegans* (***W***), and *Zea mays* (***Z***).

In Figure 7, we plot *m*_e_ (estimated number of splitting, open circle) against CDS length and fit it with a linear function (equation (10)). The observed number of splitting (closed circle) events is also plotted for comparison. The first observation from Figure 7 is that, under the GRFP model, number of splitting is linearly proportional to CDS length. On average, 78 splitting events will occur in an exon with length of 10000 bps (or around 8 events per 1000 bps). The second observation is that the observed number of splitting agrees well with the estimation from GRFP model, with the exception of non-vertebrates, especially *O. sativa*, *C. elegans*, and *D. melanogaster*.

### Parameterizing GRFP via EM iteration

In the previous simulation studies, with the assumption of known *σ*_*I*_, we have shown that *σ*_*E*_ is dependent on *α* but not on *L*_0_ and *m*, which suggests that the value of α can be estimated from *σ*_*E*_. However, simulations show that *σ*_*E*_ is also dependent on *σ*_*I*_. To derive the values of α and *σ*_*I*_ simultaneously without assuming knowing any one of them, here we determine their values through EM iterations, by combining simulations with empirically observed *σ*_*I*_ (Table 2), *μ*_*x*_, and *σ*_*x*_ (Table 3) for vertebrate genomes.

Before performing EM iteration, we need to quantify how *σ*_*I*_ is related to *σ*_*x*_. We performed a series of GRFP simulations with *σ*_*I*_ ranging from 0.06 to 0.18 and α = 3. For each simulation, we calculate the insertion ratio (equation (5)) from the resulted fragments, and estimated *σ*_*x*_ from fitting a normal function to the histogram of insertion ratios. *σ*_*x*_ is plotted against given *σ*_*I*_ in Additional file 1: Figure S5A. The plot shows that there is a linear relationship between the two. From this relationship, we estimate that real *σ*_*I*_ is closer to 0.11 than the 0.13 estimated from Figure 4. To see the over estimation of *σ*_*I*_, we show the simulation process and results in Additional file 1: Figure S6 and Additional file 1: Figure S7. By classifying adjacent exon pairs into four different groups, we show that the mixture of these four groups still follows a normal distribution but with larger *σ*_*x*_.

For EM iteration, we use *σ*_*I*_ to estimate *α*, then use estimated *α* to re-estimate *σ*_*I*_. The iteration start with *σ*_*I*_ = *σ*_*x*_ = 0.13 (Table 3).

1. Given *σ*_*I*_, determine the relationship between *α* and *σ* using simulation (Figure 6A)

2. With observed *σ*_*E*_ (Table 2) and the estimated relationship (equation (9)), estimate *α*

3. With *α*, determine the relationship between *σ*_*x*_ and *σ*_*I*_ (Additional file 1: Figure S5A)

4. With the relationship and *σ*_*x*_ = 0.13, estimate *σ*_*I*_

5. Return to step (1), iterate until convergence

The results of the EM iteration are shown in Additional file 1: Figure S5B and Additional file 1: Figure S5C. At the end, *σ*_*I*_ converges to 0.11; *α* converges to approximately 3. Again, *α* ≈ 3 suggests that, during evolution, longer exon has much higher chance to gain an intron than the shorter one, with a probability proportional to its size to the 3rd power.

### Intron losses accounting for increasing *σ*_*I*_, *σ*_*E*_ and more negative *ρ*(*i*, *j*)

In Table 3 and Figure 4, we show that *σ*_*x*_ of non-vertebrates is significantly larger than those of vertebrate genomes. In Figure 5, we also observed more negative correlation between insertion ratios for non-vertebrates. Additional file 1: Figure S1 also shows that the size distributions of non-vertebrate exons are different from those vertebrates (Figure 2). In Table 1, we have estimated that there is a significant amount of intron losses in non-vertebrate genomes. Next, numerical simulations indicate that these three differences could result from excessive intron loss during the evolution of non-vertebrate genomes.

With simulated GRFP fragments, we gradually introduce 5-50% of “intron loss” by randomly reconnecting adjacent fragment pairs. The size distributions of the resulted fragments are fitted with a normal function, (Additional file 1: Figure S8) and the fitted *σ*_*E*_ is plotted against percentage of intron loss in Additional file 1: Figure S9A. The insertion ratio between each adjacent fragment pairs is calculated using equation (5). Their histograms are fitted with a normal function (Additional file 1: Figure S10) with the fitted *σ*_*x*_ plot against intron loss in Additional file 1: Figure S9B. In Additional file 1: Figure S9C, we calculate the correlation of insertion ratios between *i* and *i*+4 sites using equation (6) for each of the intron loss simulations and plot them against intron loss. Results in Additional file 1: Figure S9 suggest that intron loss might account for increasing *σ*_*x*_ (Figure 4 and Table 3), and more negative correlation between non-adjacent insertion ratios (Figure 5).

Additional file 1: Figure S8 also shows that the size distribution of exons no longer can be fit properly to a normal function. As the percentage of intron loss increases, a second peak is appearing, as the size distribution of exons for non-vertebrates in Additional file 1: Figure S1.

## Discussion and conclusion

In this study, we analyze the size distribution of exons for 14 species, including eight vertebrates and six non-vertebrates. Our approach overcomes the limits of using orthologous genes, thus allowing us to infer evolutionary processes affecting the exon-intron structure across widely divergent species. The use of size distributions is more reliable than alignment based approaches if considering the accumulation of repeating intron gain/loss. Based on the size distribution of exons, we propose GRFP to characterize the evolution of eukaryotic genes. The solid agreements between GRFP simulations and observations on genomic data provide several key findings on the evolution of exon-intron gene structures.

### Chance of intron gain is proportional to exon size to the 3rd power

GRFP reveals that longer exons have a higher chance to gain an intron during evolution, and reveals the novel finding that the chance of intron gain is proportional to the exon length to the third power. This finding was derived after investigating real genome data, comparing with numerical simulations, and excluding various effects on GRFP through EM iterations. This finding might explain why long exons are rare in modern organisms. E.g., statistical study has shown that only 3.5% of the primate exons are longer than 300 nt [8,9,25].

The “third power” is derived from *σ*_*E*_ (or width) of the exon size distributions. The model of GRFP indicates that *σ*_*E*_ will remain constant given the same dependency value *α*. Thus, the nearly identical *σ*_*E*_ (0.43) in Table 2 suggests the existence of a common dependency value (*α* ≈ 3) across vertebrate species. However, the 5′ deviations of *α* value might indicate that intron is less preferred there. For non-vertebrate species, *α* cannot be directly estimated since their exons follow a mixture of two lognormal distributions (instead of one) possibly due to excessive intron loss. For estimation of intron loss in Table 1, we simply assume that *α* ≈ 3 holds for non-vertebrates.

Why is the probability of intron gain proportional to the exon length to the third power? Given that the third power is usually related to volume, it might be possible that exon occupies a volume proportional to its length to the third power due to dynamic movement, and the chance of an intron attacking it is proportional to this volume. Further investigation will be needed to support this hypothesis.

### No evidence for site-specific bias of intron insertion

We derive this finding from indirectly estimating the position distribution of intron insertion loci. We demonstrate that the insertion loci follow a normal distribution, peaking around the center of the exon with a standard deviation (*σ*_*I*_) of 0.11. This observation does not support the proto-splice site hypothesis. If there were proto-splice sites in the exon, the insertion loci would follow the position distribution of these sites, which will most likely be a uniform distribution (Additional file 1: Figure S3).

In Figure 5, we also demonstrate that, for vertebrate genomes, intron gains are independent of each other. This observation is also one of the core assumptions of GRFP simulation. It holds on vertebrate genomes and non-vertebrate genomes if the effect of intron losses is considered. Another simplification of GRFP is that the effect of exon duplications is ignored. As mentioned earlier, the sharp spikes in Figure 4 are related to tandem exon duplication [22]. Such effect is not considered since overall their contribution is not significant in estimating either insertion loci or the chance of intron gain. This is illustrated in Additional file 1: Figure S7 and Additional file 1: Figure S10, where no such spikes are observed.

The assumption behind the estimation of insertion ratio (equation (5)) is that the order of the exons within each gene is maintained during evolution. In the cases of tandem exon duplication, exon shuffling, or intron loss, the order is just locally disrupted. Simulation also shows that the estimated insertion ratio is a mixture of four different groups of adjacent fragment pairs (Additional file 1: Figure S7, Additional file 1: Figure S8), but *σ*_*x*_ is linearly related to *σ*_*I*_ (Additional file 1: Figure S5A).

### Suggesting 5′ intron gain/loss bias

By grouping exons by positions within a gene, we demonstrate that exons next to the 5′ UTR have bigger standard deviation (*σ*_*E*_) than other exons. One may argue that the deviation near the 5′ UTR is caused by the fact that on average exons are longer for genes contain fewer exons. If this is the case, similar trend near the 3′ UTR should have been observed. From equation (9), bigger *σ*_*E*_ indicates smaller GRFP dependency value (*α*). The dropping of *α* values for exons adjacent to the 5′ UTR implies that introns are not favored there; In the GRFP model (Equation (1)), a smaller dependency value indicates a lower chance in acquiring introns during evolution. Alternatively, it might be explained as intron loss bias, that is, introns right after 5′ UTR has a tendency to lose than other introns. Certainly such comparison is limited to introns in the coding region and debatable due to the unclear stochastic process of intron loss.

### Excessive intron losses accounting for deviations from GRFP

In this study, we show that exons of non-vertebrates are different from those of vertebrates in three aspects. First, the size distribution of their exons fit a mixture of two normal distributions (Additional file 1: Figure S1) instead of one for vertebrates (Figure 2). Second, their insertion ratios have much larger standard deviations (*σ*_*x*_) as shown in Table 3. Third, their non-adjacent insertion ratios are more negatively correlated as shown in Figure 5.

The estimations in Table 1 (also Figure 7) suggest that there are excessive intron losses in non-vertebrate genomes. Based on this, we performed simulations of intron loss after GRFP fragmentation. Additional file 1: Figure S9 demonstrates that, with increasing intron losses, *σ*_*E*_ increases, *σ*_*I*_ increases, and *ρ*(*i*, *j*) decreases from zero, consistent with the observation on empirical data analysis here. Therefore, GRFP model holds on non-vertebrate genomes when the effect of intron loss is considered. Comparative approaches also show that frequent intron loss has been inferred during the evolution of Nematode [26,27] and Drosophila genomes [28], though they cannot provide a genome wide estimation of percentage of intron losses.

Here, the excessive intron loss hypothesis in non-vertebrate genomes is interpreted as breaking the equilibrium between intron gain and intron loss. Although GRFP model is built on modeling intron gain events, it does not assume that introns in vertebrate genomes are never lost. Instead, we interpret the straight line in Figure 7 as a “dynamic equilibrium line for vertebrates”, where the genome reaches a state of stability for its intron count. In Additional file 1: Figure S11, we observed the similar linear relationship between intron counts and CDS length by grouping genes by chromosomes for *H. sapiens*. This further proves that the “equilibrium” is reached in each chromosome and the linear relationship is independent of lineage (since human chromosomes are not related by a simple lineage relationship). If there are many intron losses during evolution, subsequent gains must have also occurred to bring the genome back to the equilibrium line. Therefore, the statistical measurements of the exons can remain the same across examined vertebrate genomes. For the non-vertebrate genomes that fall below the line, equilibrium is shifted to intron loss relative to vertebrates. The shifting (variation of intron density) might be related to the differences in the generation time of each species [29].

The size distribution of exons (Additional file 1: Figure S1), the insertion ratios (Table 3) and the correlation map (Figure 5) suggest that *A. thaliana*, *S. bicolor*, and *Z. mays* underwent intron loss during evolution though not as significant as *O. sativa*. The estimated intron losses are around 7% for *A. thaliana* and *S. bicolor*, and 4% for *Z. mays* (Table 1). However, such percentage of intron loss is not significant enough to justify the size distribution of exons for these three plant species, a mixture of two Gaussians instead of one as shown in Additional file 1: Figure S1. Another possible explanation is that plants have undergone significant genome duplications and the rate of indels is higher for the duplicate genes [30]. The reason is that one copy of the duplicate genes is freed from the selection pressure.

### Weakness and strength of GRFP

In this work, we propose the GRFP model to capture the dynamic processes describing the evolution of exon-intron structures. For vertebrate genomes, the model fits well with the well annotated genome data, including exon size distribution, distribution of insertion loci, total CDS length, number of introns, independency among intron gains, and 5′ intron gain bias. For non-vertebrate genomes, simulations show that the deviations from the vertebrate genome can be explained by excessive intron loss. The GRFP model implies that the evolution of gene structure is purely random, from picking which exon to split (gains intron) to picking intron insertion loci on the selected exon. The solid agreements between GRFP simulations and real genome data confirm that GRFP model provides one possible explanation on the exon-intron structure evolution.

It is well known that a modern genome is a collection of introns that have accreted (and been deleted) over at least a billion years. Here, by considering the whole process as a black box, we reproduce the output of this box (the current day genomes) with numerical simulations. The size distribution of exons serves as the key component in building GRFP model because of two reasons. First, the dominant factor that can shape such distribution is intron gain/loss (fragmentation). Second, the most prominent cofounding factor on exon sizes - the rate of indels in them during evolution is low. Certainly, the mechanism of intron gain is complicated considering differences across lineage, differences in rates of insertion across sites, the age of introns, the possibility of indels to maximize fit to epigenomic structures that can occur following intron gain, alternative splicing, the different models of intron gain, and so on. The process of exon fragmentation (or intron gain) might be as straightforward as the model of GRFP describes. By focusing on internal translated exons only, we have demonstrated outstanding agreements between empirical observations and GRFP simulations.

It is crucial to note that GRFP does not make any assumptions on the rate of intron gain/loss. Recent studies [6,7,31-36] have suggested that the early eukaryotes experienced a rapid burst of intron gain. In later evolution, intron loss dominates the landscape, with occasional bursts of intron gains. This does not contradict the results presented here since GRFP makes no assumptions about the rate of intron gain or loss during evolution, but instead estimates the chance for an exon to gain an intron somewhere along its length and predicts the distribution of those insertion events.

One may argue that the agreement between the GRFP model and well annotated genome structure could be fortuitous. While we cannot rule out that other models might reproduce the exons of modern genomes, the predictive power of GRFP is striking, and we believe that it is a promising approach to understanding the evolution of exon-intron structures, and an excellent starting point for new models for revealing the hidden stochastic processes of evolution.

### Unanswered questions and future studies

GRFP model provides explicit rules on the exon-intron structure evolution. However, it does not address the origin of introns, the mechanism of intron insertion, and the rate of intron gain/loss. In other words, GRFP addresses where introns are inserted (which exon and where in the exon), but not when and how introns are inserted. Future research will focus on extending GRFP to model the evolution of noncoding exon, and developing GRFP-based methods for comparative genomics studies.

## Abbreviations

GRFP: General Random Fragmentation Process; UTR: Untranslated region; CDS: Coding DNA Sequence; EM: Expectation Maximization.

## Competing interests

The authors declare that they have no competing interests.

## Authors' contributions

LW developed the model, performed the data analysis and designed the simulation experiment. LW and LDS wrote the manuscript. All authors read and approved the final manuscript.

## Supplementary Material

Additional file 1: Figure S1 has the size distribution of non-vertebrate exons. **Figure S2** has the size distributions of *H. sapiens* exons grouped by position, supporting the plot in Figure 3. **Figure S3** shows that the distribution of proto-splice sites within *H. sapiens* coding sequences is uniform. **Figure S4** shows the size distribution of simulated exons with different dependency values. **Figure S5** shows the linear relationship between expected and observed standard deviation of insertion ratios. **Figure S6** illustrates four different groups of insertion ratios. **Figure S7** shows the distribution of insertion ratio for each of the four groups and their mixture. **Figure S8** shows the distribution of fragment size after a certain percentage of intron losses, supporting **Figure S9A**. **Figure S10** shows the distribution of insertion ratios after a certain percentage of intron loss, supporting **Figure S9B**. **Figure S11** shows the linear relationship between the number of splitting and total CDS length for each *H. sapiens* chromosome. Click here for file
