# Supplementary material for: Modeling the evolution dynamics of exon-intron structure with a general random fragmentation process
Source: BMC Evol Biol. 2013 Feb 28;13:57. doi: 10.1186/1471-2148-13-57 (PMC3732091; doi:10.1186/1471-2148-13-57)

Figure S1. **Size distributions of non-vertebrate itexons fitting with a mixture of two normal distributions.**
The histograms of itexons are fitted with a mixture of two normal functions (dashed line). Solid line is the summation of these two normal functions.

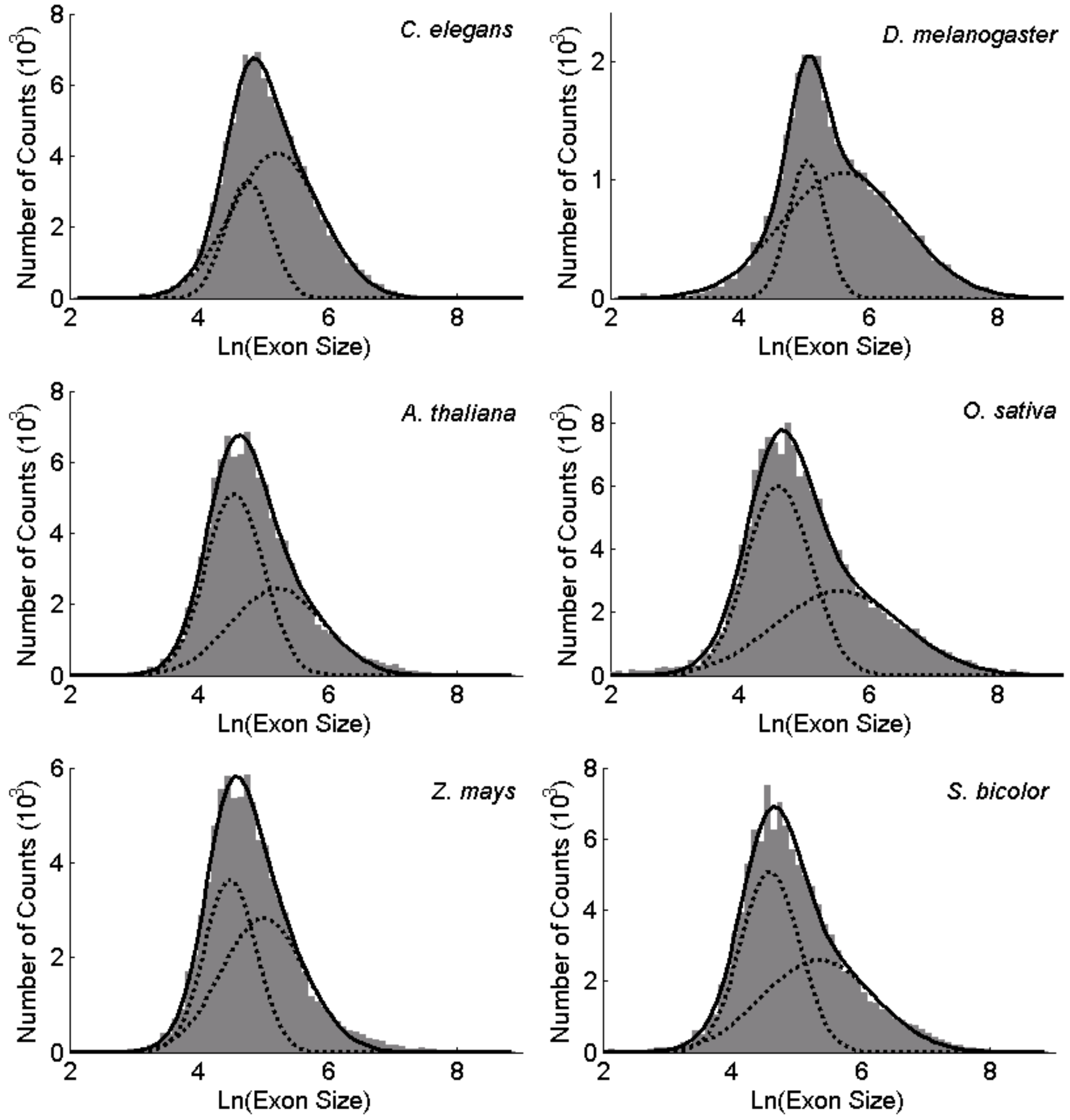

Figure S2. Size distribution of *H. sapiens* itexons grouped by position (top right corner) relative to 3′ UTR (left) or 5′ UTR (right). Bin size of the histograms is 0.1. Each histogram is fitted with a Weibull (solid line) and a normal function (dashed line) separately.

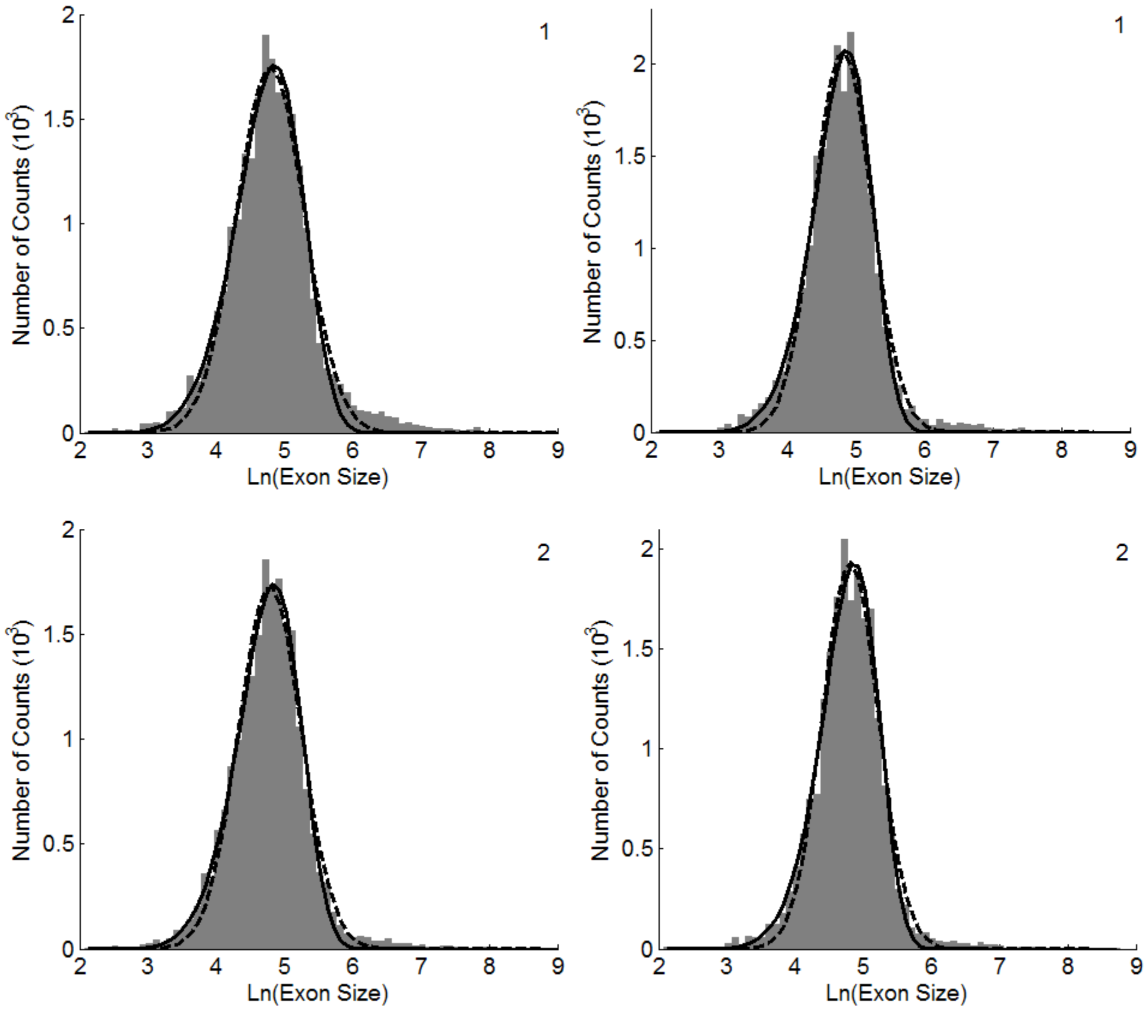

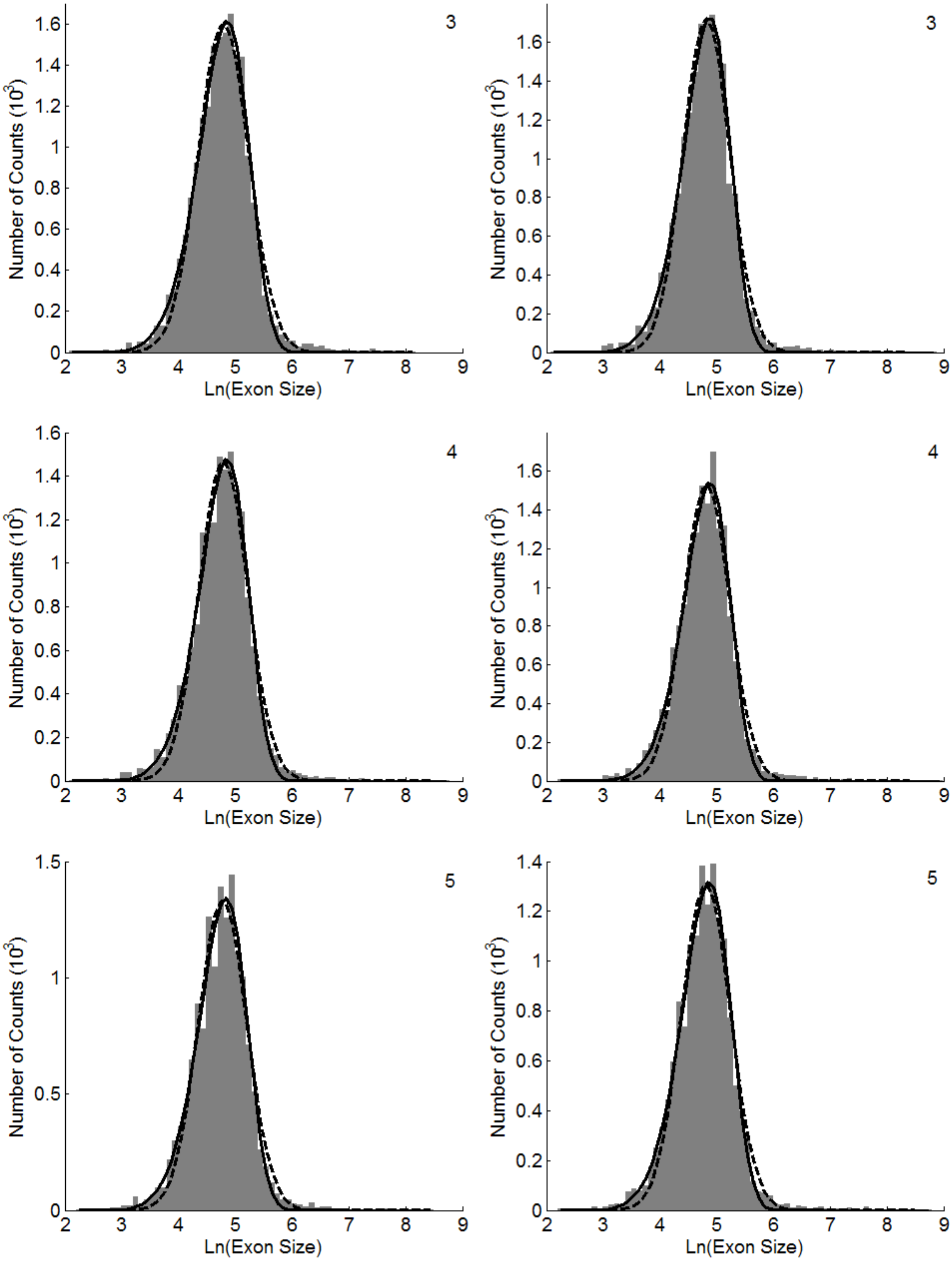
3
3
4
4
5
5
Number of Counts (10^3)
Ln(Exon Size)
0
0.2
0.4
0.6
0.8
1
1.2
1.4
1.6
0.5
1.5
2
3
4
5
6
7
8
9

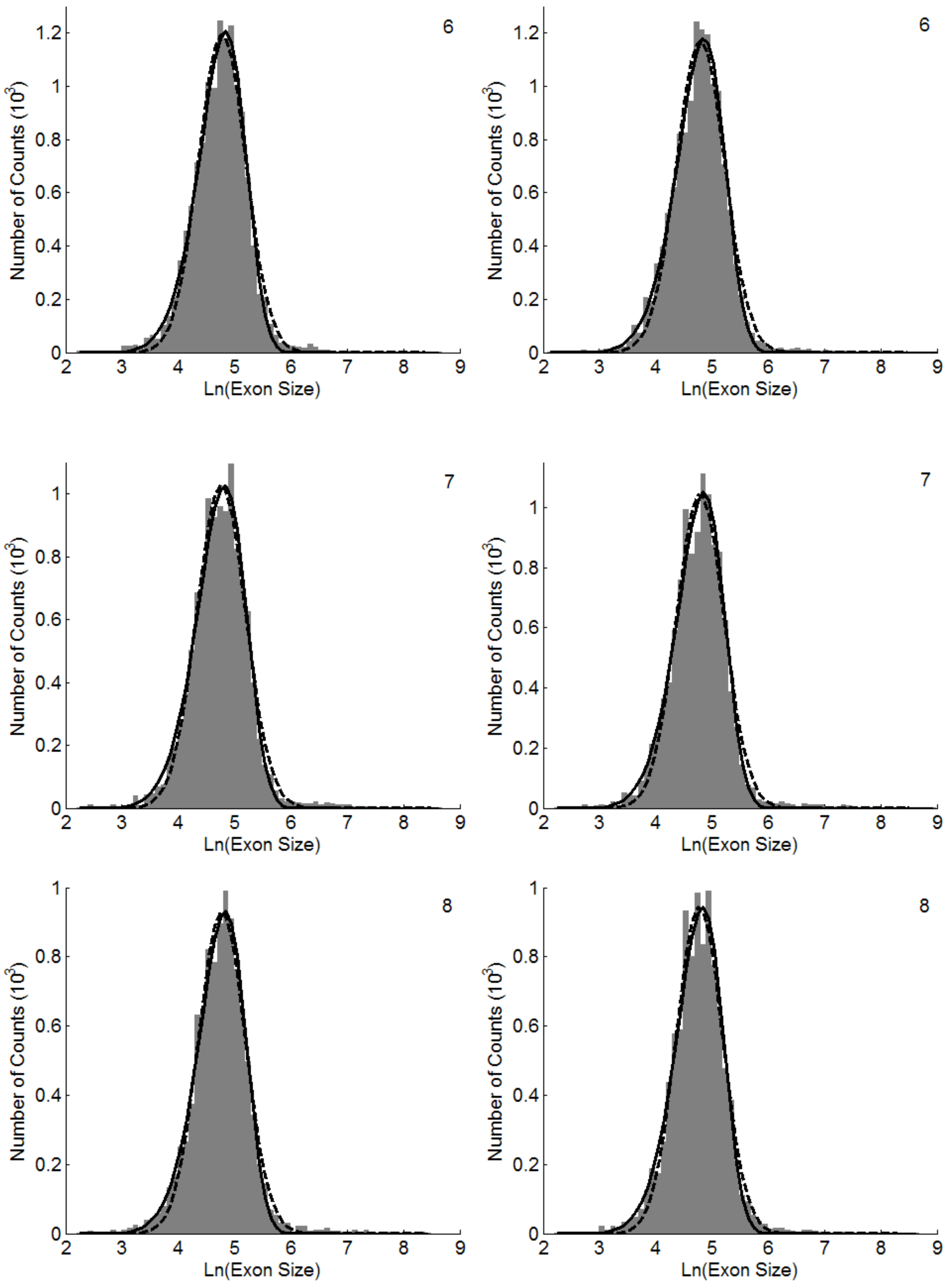
6
Number of Counts (10^3)
Ln(Exon Size)
6
Number of Counts (10^3)
Ln(Exon Size)
7
Number of Counts (10^3)
Ln(Exon Size)
7
Number of Counts (10^3)
Ln(Exon Size)
8
Number of Counts (10^3)
Ln(Exon Size)
8
Number of Counts (10^3)
Ln(Exon Size)

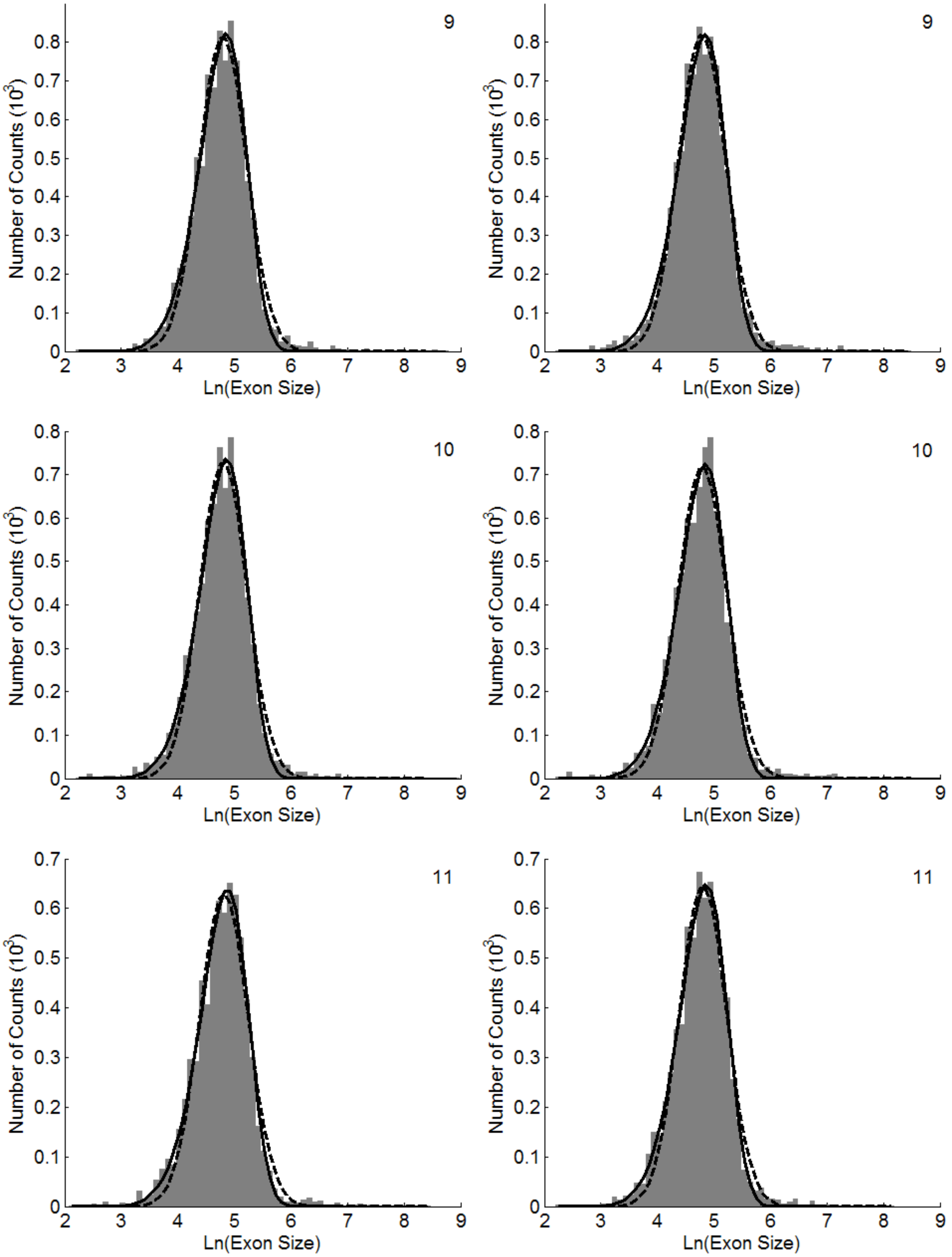
9
9
10
10
11
11
Number of Counts (10^3)
Ln(Exon Size)

Figure S3. Distributions of proto-splice sites within *H. sapiens* coding sequences.

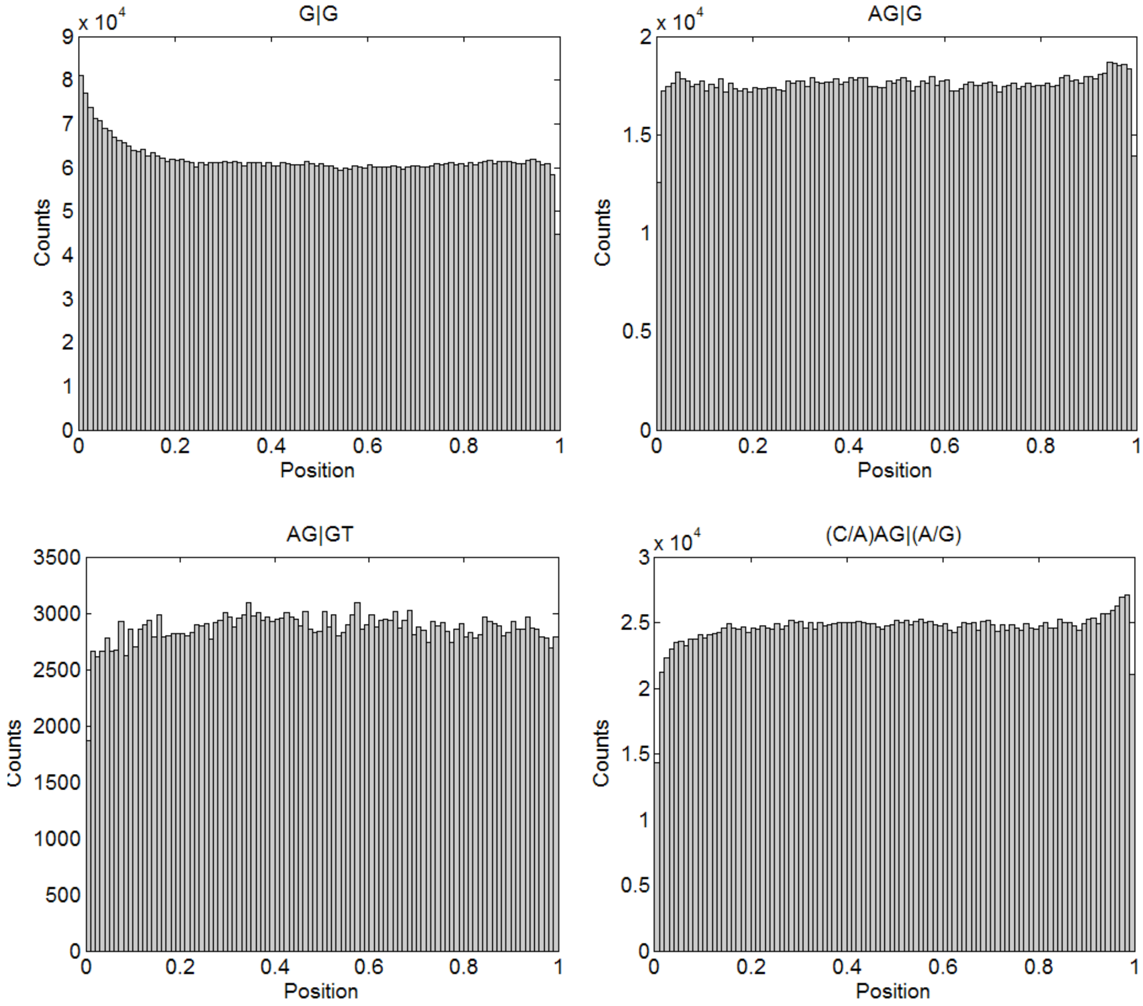

Figure S4. **Size distributions of the fragments from GRFP simulations with different length dependency (α).**
(A) α = 0.3; (B) α = 1; (C) α = 3.. Solid (dashed) lines show the fitting with a Weibull (normal) function. Bin size is 0.1.

A

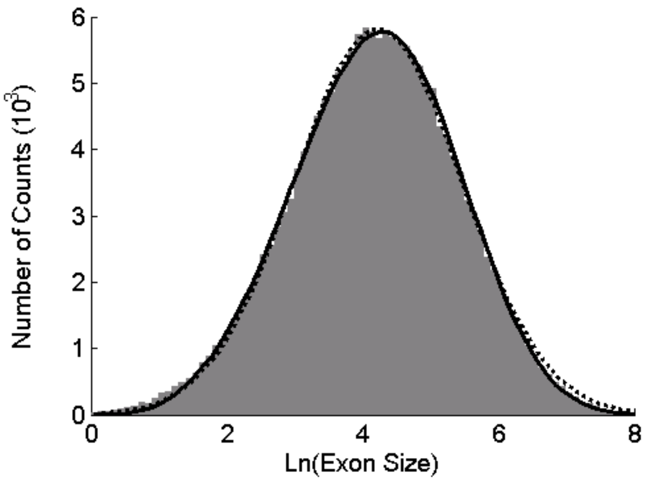

B

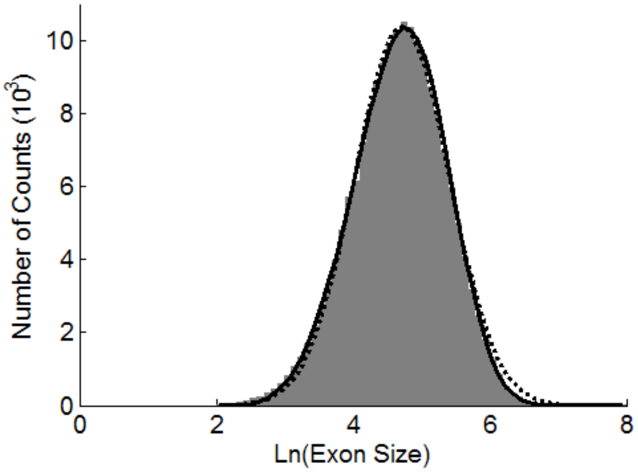

C

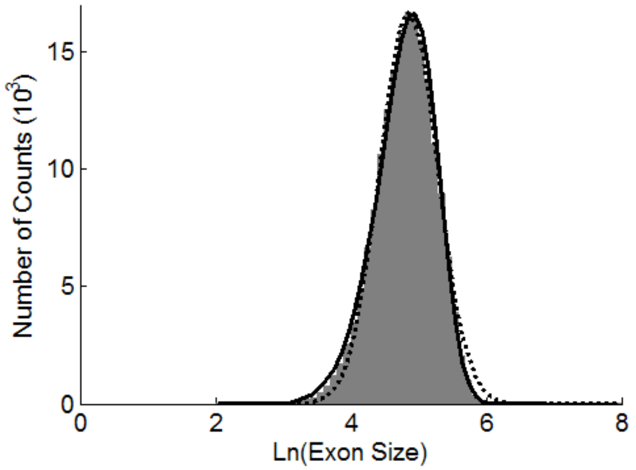

Figure S5. EM iterations for determining α and σ_I .

(A) Empirical relationship between given insertion standard deviation (σ_I) and the estimated σ_x . (B) Convergence of α and σ_I **.**

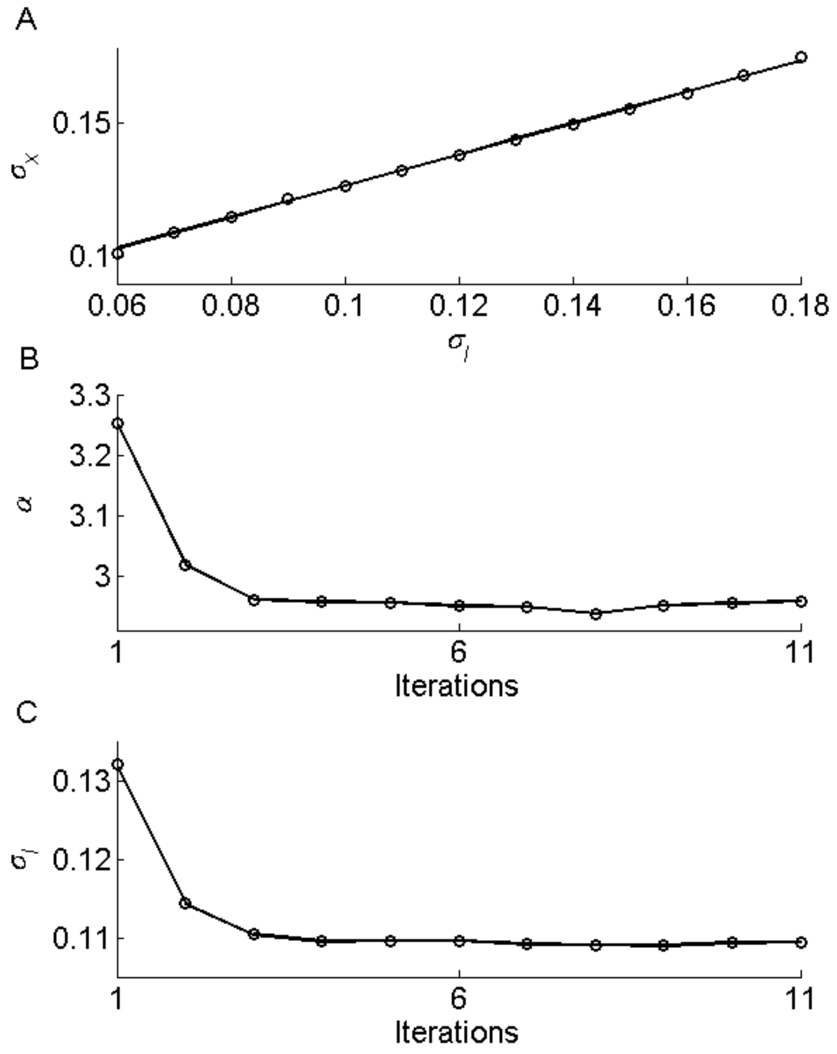

Figure S6. Illustration of intron insertion given initial exon size 1 (first row). At the end, six introns are inserted to split the single exon into seven exons (fourth row). The length of each exon in the diagram is labeled. For demonstration of commonality, in the third row, the third exon from the left does not split. The insertion ratios, as shown in equation (8), are calculated for each adjacent exon pairs in the fourth row and shown below the diagram. If considering the intron gains/splitting process as a tree, the six ratio values can be classified into four different groups based on depth and parent of the adjacent exons.

- Group x, including x_4, x_5, and x_6, calculated from two adjacent exons with same depths and parents
- Group y_1, including just y_1, calculated from two adjacent exons with the same depth but different parents
- Group y_2, including just y2, calculated from two different exons with different depth (left_depth > right_depth)
- Group y_3, including just y3, calculated from two different exons with different depth (left_depth < right_depth)

We can infer the distribution of insertion loci from group x ratios if we know where they are. In reality, we can only calculate ratios from any two adjacent itexon pairs, which give us a mixture of these four groups.

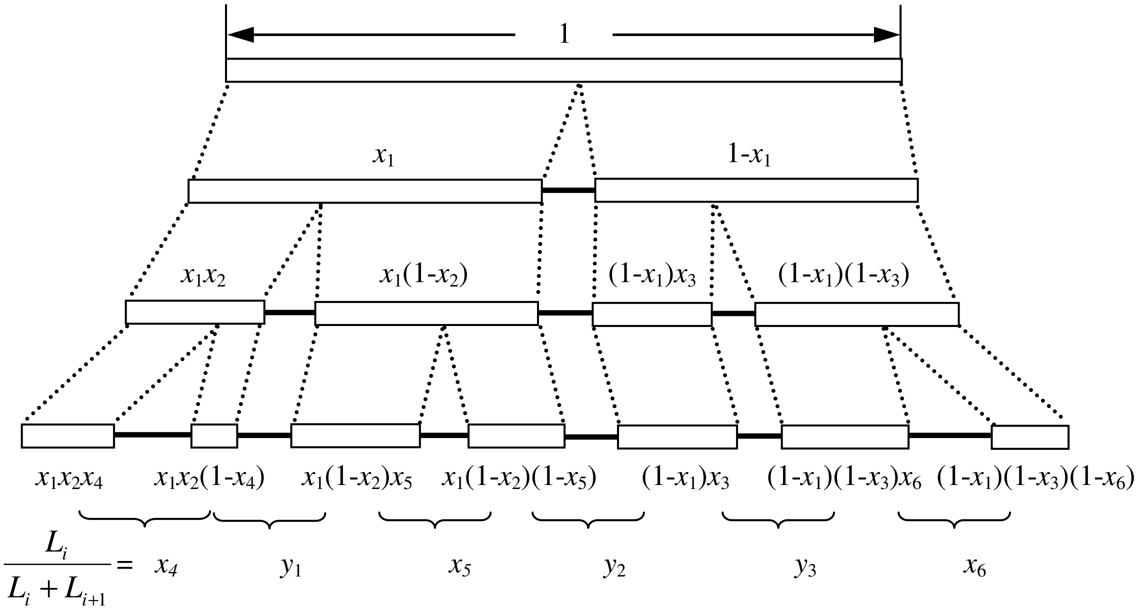

$$y_1 = \frac{1}{1 + \frac{x_5(1 - x_2)}{x_2(1 - x_4)}} \qquad y_2 = \frac{1}{1 + \frac{x_3(1 - x_1)}{x_1(1 - x_2)(1 - x_5)}} \qquad y_3 = \frac{1}{1 + \frac{(1 - x_3)x_6}{x_3}}$$

Figure S7. Histogram of x, y_1, y_2, y_3 and their union. The histogram is drawn with bin size of 0.01 and fit with normal distribution. Fitted mean and standard deviation are shown in parenthesis. The GFRP simulation starts with initial size of 10^{11}, $\alpha = 3$, σ_I = 0.11, and stops after 10^5 splitting. The insertion ratios from adjacent exon pairs are grouped by the depth and parents of the two exons (as described in Figure S3). The union does distribute normally with μ_I = 0.5 and σ_I = 0.13, bigger than the given value of 0.11.

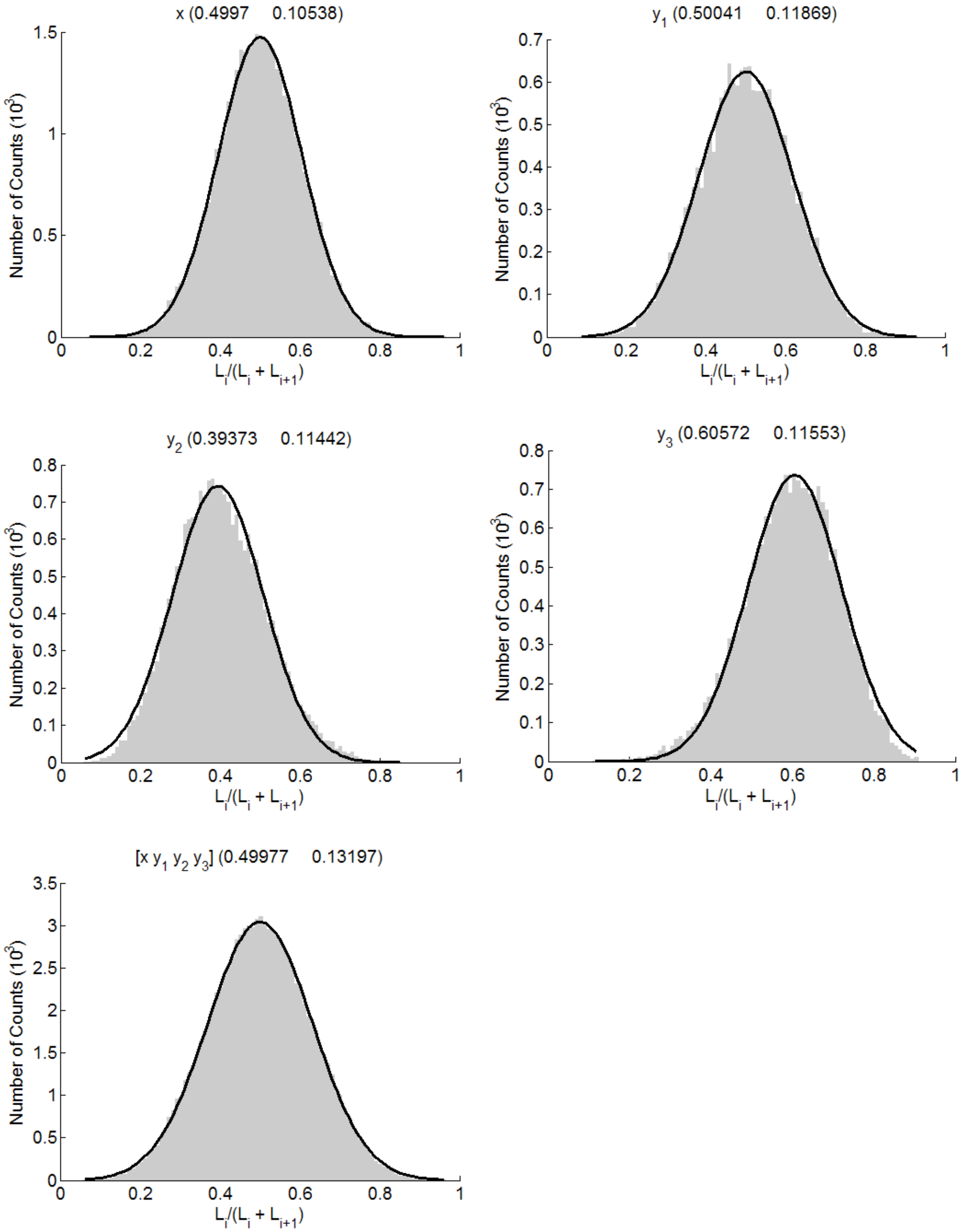

Figure S8. Distributions of fragment size after certain percentages of intron loss. GRFP simulation starts with L_0=10^{11}, m=10^4, then different percentages of intron loss (5%, 10%, 20%, and 30%) are introduced and the resulted size distributions are shown below. The histograms are fitted with a normal function with σ shown on top.

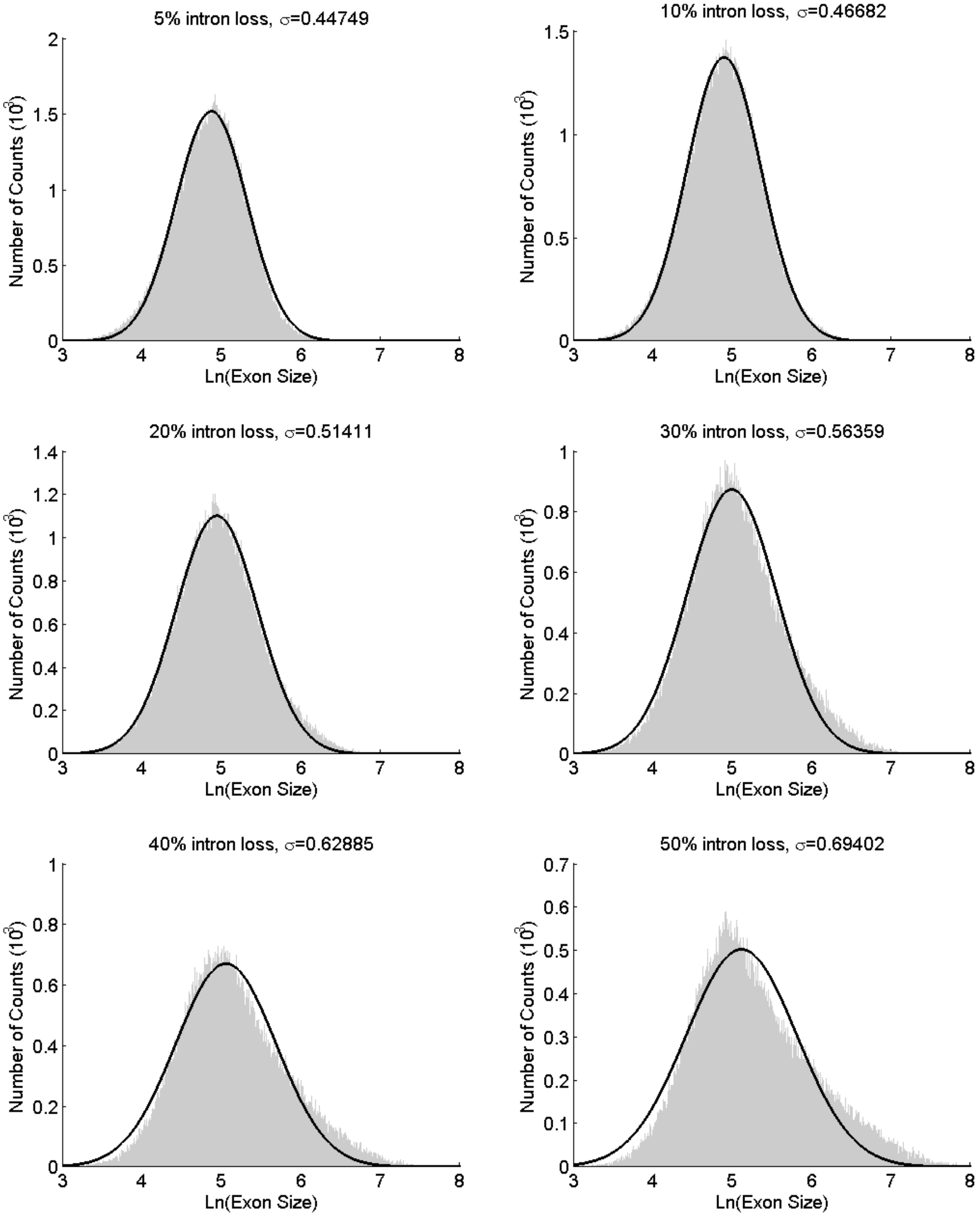

Figure S9. The effects of intron loss on σ_E , σ_x , and $\rho(i,\ j)$.

(A) σ_E as a function of intron loss. (B) σ_x as a function of intron loss. (C) $\rho(i,\ i+4)$ as a function of percentage of intron loss.

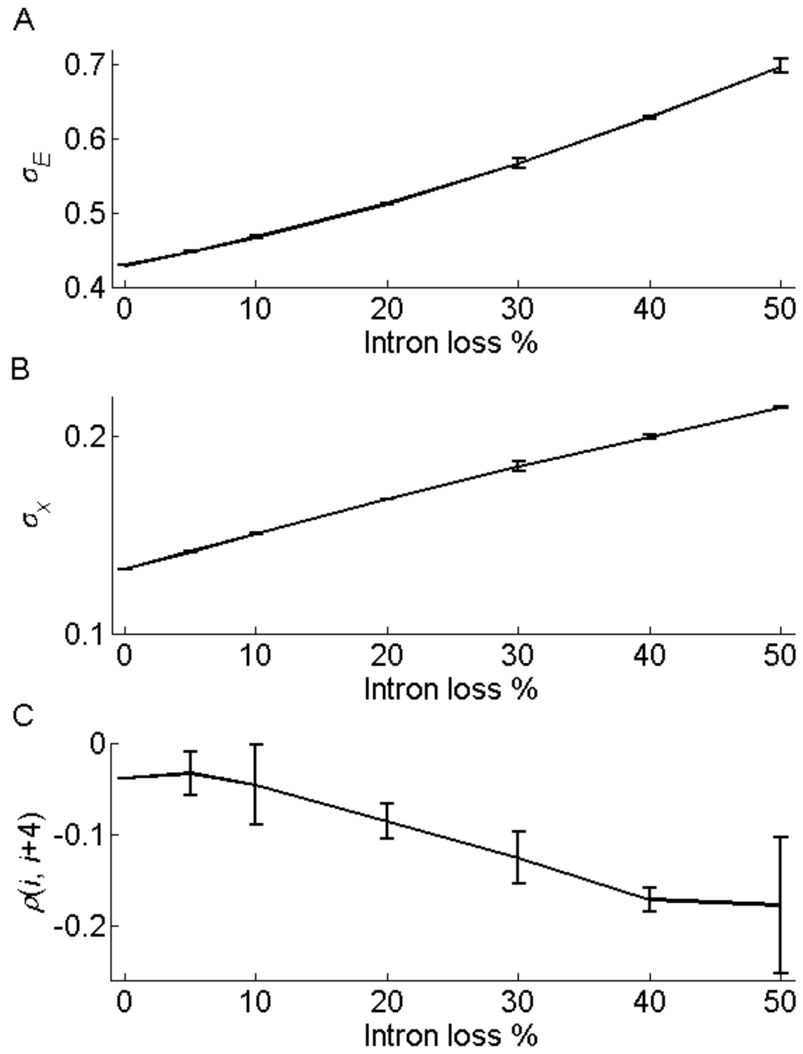

Figure S10. Distributions of insertion ratios after certain percentages of intron loss. The histograms are fitted with a lognormal function with the standard deviation (σ_I) shown on top of the plot.

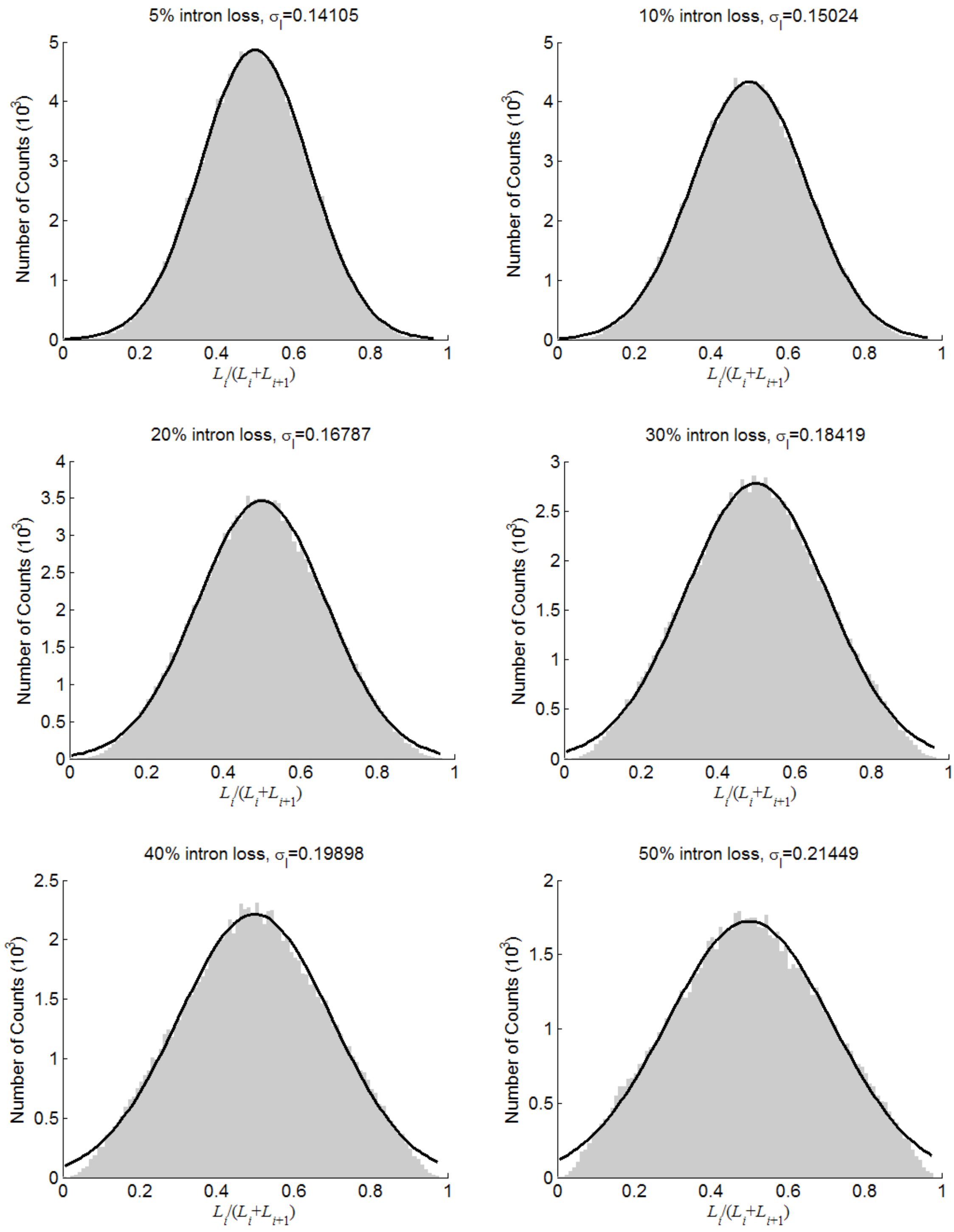

Figure S11. **Plot of the number of splittings as a function of total CDS length for each H. sapiens chromosome.**
The observed splitting (open circle) is plotted with CDS length in different chromosomes (23 is X chromosome, and 24 is Y chromosome), and fitted with a linear function (solid line with a slope of 0.0074 and an intersection value of 96).

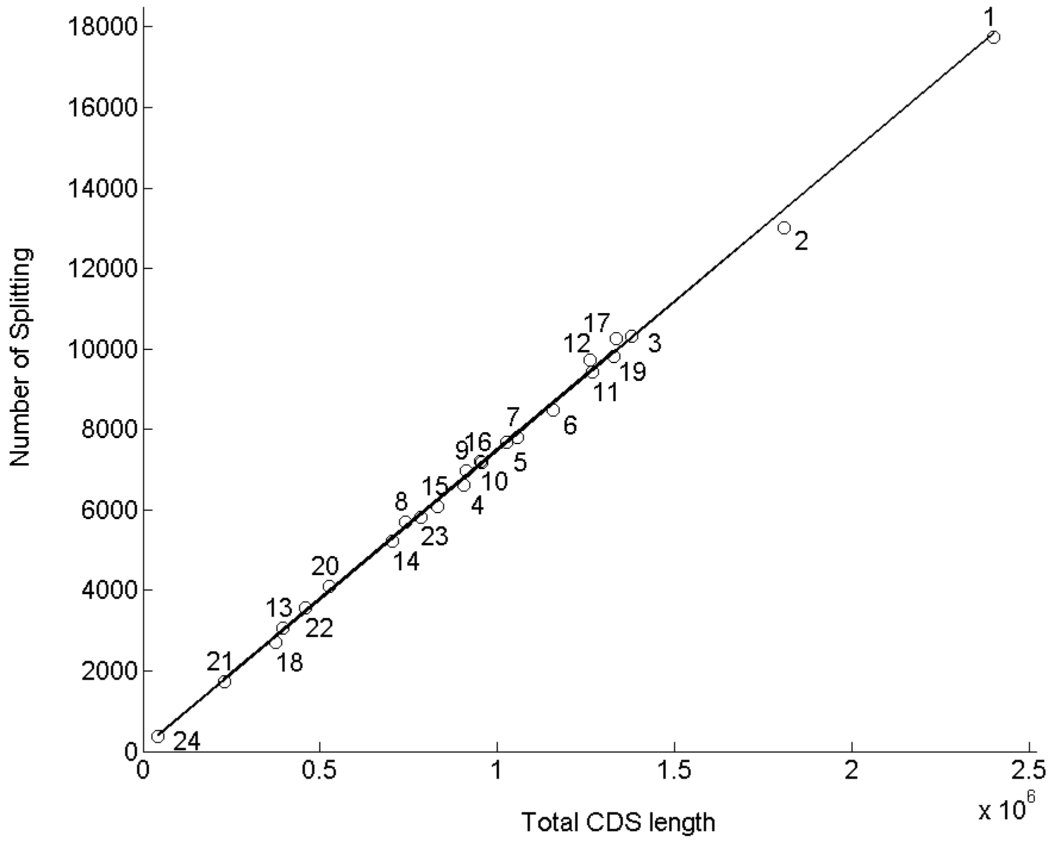

Supplement: Additional file 1: Figure S1 — has the size distribution of non-vertebrate exons. Figure S2 has the size distributions of H. sapiens exons grouped by position, supporting the plot in Figure 3. Figure S3 shows that the distribution of proto-splice sites within H. sapiens coding sequences is uniform. Figure S4 shows the size distribution of simulated exons with different dependency values. Figure S5 shows the linear relationship between expected and observed standard deviation of insertion ratios. Figure S6 illustrates four different groups of insertion ratios. Figure S7 shows the distribution of insertion ratio for each of the four groups and their mixture. Figure S8 shows the distribution of fragment size after a certain percentage of intron losses, supporting Figure S9A. Figure S10 shows the distribution of insertion ratios after a certain percentage of intron loss, supporting Figure S9B. Figure S11 shows the linear relationship between the number of splitting and total CDS length for each H. sapiens chromosome. [file 1471-2148-13-57-S1.pdf]
